# Meningeal Foam Cells and Ependymal Cells in Axolotl Spinal Cord Regeneration

**DOI:** 10.3389/fimmu.2019.02558

**Published:** 2019-11-01

**Authors:** Nathaniel Enos, Hidehito Takenaka, Sarah Scott, Hai V. N. Salfity, Maia Kirk, Margaret W. Egar, Deborah A. Sarria, Denise Slayback-Barry, Teri Belecky-Adams, Ellen A. G. Chernoff

**Affiliations:** Department of Biology, Indiana University-Purdue University Indianapolis, Indianapolis, IN, United States

**Keywords:** foam cells, foamy macrophages, ependymal cells, meningeal fibrosis, spinal cord regeneration, axolotl regeneration, lipid uptake, myelin uptake

## Abstract

A previously unreported population of foam cells (foamy macrophages) accumulates in the invasive fibrotic meninges during gap regeneration of transected adult Axolotl spinal cord (salamander *Ambystoma mexicanum*) and may act beneficially. Multinucleated giant cells (MNGCs) also occurred in the fibrotic meninges. Actin-label localization and transmission electron microscopy showed characteristic foam cell and MNGC podosome and ruffled border-containing sealing ring structures involved in substratum attachment, with characteristic intermediate filament accumulations surrounding nuclei. These cells co-localized with regenerating cord ependymal cell (ependymoglial) outgrowth. Phase contrast-bright droplets labeled with Oil Red O, DiI, and DyRect polar lipid live cell label showed accumulated foamy macrophages to be heavily lipid-laden, while reactive ependymoglia contained smaller lipid droplets. Both cell types contained both neutral and polar lipids in lipid droplets. Foamy macrophages and ependymoglia expressed the lipid scavenger receptor CD36 (fatty acid translocase) and the co-transporter toll-like receptor-4 (TLR4). Competitive inhibitor treatment using the modified fatty acid Sulfo-N-succinimidyl Oleate verified the role of the lipid scavenger receptor CD36 in lipid uptake studies *in vitro*. Fluoromyelin staining showed both cell types took up myelin fragments *in situ* during the regeneration process. Foam cells took up DiI-Ox-LDL and DiI-myelin fragments *in vitro* while ependymoglia took up only DiI-myelin *in vitro*. Both cell types expressed the cysteine proteinase cathepsin K, with foam cells sequestering cathepsin K within the sealing ring adjacent to the culture substratum. The two cell types act as sinks for Ox-LDL and myelin fragments within the lesion site, with foamy macrophages showing more Ox-LDL uptake activity. Cathepsin K activity and cellular localization suggested that foamy macrophages digest ECM within reactive meninges, while ependymal cells act from within the spinal cord tissue during outgrowth into the lesion site, acting in complementary fashion. Small MNGCs also expressed lipid transporters and showed cathepsin K activity. Comparison of ^3^H-glucosamine uptake in ependymal cells and foam cells showed that only ependymal cells produce glycosaminoglycan and proteoglycan-containing ECM, while the cathepsin studies showed both cell types remove ECM. Interaction of foam cells and ependymoglia *in vitro* supported the dispersion of ependymal outgrowth associated with tissue reconstruction in Axolotl spinal cord regeneration.

## Introduction

This research examines a previously unidentified phenomenon in spinal cord regeneration involving the accumulation of foamy macrophages in fibrotic meninges during amphibian spinal cord regeneration. In the transected spinal cord, during gap regeneration, of the adult Axolotl (an aquatic salamander, *Ambystoma mexicanum*) foamy macrophages took up lesion site lipids and myelin while degrading ECM. These innate immune system cells were also found in close association with Axolotl ependymal cell (ependymoglial) outgrowth that remodels the cord following injury. The interaction of foamy macrophage with ependymal cells *in vitro* modified ependymal cell behavior related to mesenchymal outgrowth.

The role of ependymal cells has been investigated extensively in urodele spinal cord regeneration. Studies include ependymal growth factor and retinoid responses, ECM formation and removal, cytoskeletal changes, remodeling of radial processes and epithelial to mesenchymal transition, association with axonal outgrowth, stem cell properties and neurogenesis, and dorsal-ventral patterning of the regenerating cord (1–17). The role of a meningeal reaction in urodele spinal cord regeneration has a far less extensive body of work (5, 12, 13). The present research explores aspects of the urodele spinal meninges response complementary to the earlier studies.

Meningeal fibrosis occurs after penetrating spinal cord injury (SCI) in urodele amphibians (newts and salamanders), as it does in mammals [rev. (10, 15)]. Penetrating mammalian SCI induces a meningeal (fibrotic) scar that inhibits axonal regrowth directly and reinforces the astrocytic (gliotic) scar (18, 19). This dual scarring process forms a permanent barrier to axonal regrowth. In urodeles, fibrotic meninges is remodeled and excluded to the periphery of regenerating cord, a process that involves ependymal outgrowth and digestion of extracellular matrix (10, 12, 15, 20). Stensaas (5) and Zukor et al. (12) showed an intimate association of reactive meninges with multiple cell types in transected newt spinal cord. Reactive newt meninges and cord outgrowth were shown to contain macrophages that contact regenerating neurons and ependymoglia during the regenerative process (12).

Foamy macrophages, also known as foam cells, foamy phagocytes or foamy histiocytes, are of monocyte origin and distinguished by the “foamy” appearance of their extensive lipid inclusions in histological preparations (21, 22). They can fuse into “osteoclast-like” MNGCs (21, 23, 24). Foamy macrophages can serve as sinks for lipoproteins and myelin fragments in pathological neural conditions, such as multiple sclerosis (21, 25–27). They can be, at least transiently, beneficial in this pathology (22, 27).

Foamy macrophages form from monocyte-derived M2-macrophage (anti-inflammatory macrophage) precursors (26, 28, 29). Features of foam cells *in vivo* and *in vitro* include: clusters of lipid inclusions that are phase contrast bright, stain with Oil Red O or the indocarbocyanine dye DiI, production of the cysteine proteinase cathepsin K, activity of the lipid scavenger receptor CD36, uptake of oxidized low density lipoprotein (Ox-LDL), and uptake of myelin fragments. These features are characteristic of live cell lipid droplets, foam cells and osteoclast-like MNGCs derived from foam cells (21, 25–27, 30–33).

In mammalian SCI, foamy macrophages form only within injured spinal cord tissue, where they take up myelin and contribute to a pro-inflammatory environment (34). Accumulation of foamy macrophages has not been shown within injured mammalian spinal meninges (34, 35). Macrophages have been described within injured salamander spinal cord, as well, and many immune responsive genes are upregulated shortly after Axolotl SCI (12, 36, 37). However, foamy macrophages have not previously been reported in salamander cord or meninges.

Uptake of the toxic lipid metabolites after neural injury can be approximated *in vitro* by uptake of Ox-LDL (38). A common lipid transport mechanism involved in the uptake of Ox-LDL uses CD36, a class B scavenger receptor/fatty acid translocase (25, 39). In atherosclerosis and other pathological conditions, CD36 and Toll-like Receptor-4 (TLR4), along with TLR6, act together in lipid uptake and inflammatory behavior (40). CD 36 is also involved in fusion of macrophages to form MNGCs (23, 24). These studies suggest the use of an Ox-LDL uptake model and examination of the role of CD36 in Axolotl meningeal foam cell lipid transport.

In many neural pathologies, foamy macrophages and MNGCs also take up myelin sheath products by phagocytosis. Myelin debris persists for extended periods in mammalian spinal cord lesion sites and is sequestered in macrophages (41, 42). Extensive myelin fragment uptake by foamy macrophages occurs within active and chronic-active plaques in the CNS in multiple sclerosis (25–27, 43). In animal models of amyotrophic lateral sclerosis, foamy macrophages are involved in myelin uptake during Wallerian degeneration in the peripheral nerves, associated with loss of axons and neuromuscular synapses (44–46). In Charcot-Marie-Tooth disease, a group of peripheral nervous system (PNS) demyelinating disorders, foamy macrophages with myelin inclusions are found next to poorly myelinated or demyelinated axons (47). Foamy, myelin-containing macrophages are also found in association with peripheral nerve degeneration in aging mice (48). In some of these pathologies the literature is contradictory on the pro-inflammatory or anti-inflammatory nature of the foam cells involved in this processes, depending on the type of experimental system, stage of disease or the markers examined (27, 46, 47, 49–51). The question whether these foam cell effects are beneficial or harmful is even more complex. The work presented here showed uptake of myelin *in situ* and *in vitro* similar to that in seen in mammalian neural pathologies.

A third critical property of foamy macrophages in the nervous system, in addition to oxidized lipoprotein and myelin uptake, is their ability to degrade ECM (32). This process appears to be universal in foamy macrophages and occurs in osteoclast-like MNGCs associated with atherosclerosis and MNGC tumors (52, 53). Foamy macrophages and MNGCs digest ECM using cathepsin K and MMP9 as major secreted proteolytic factors (32, 52–55). Secreted proteases can be concentrated on the matrix within the sealing ring, a set of cytoskeletal and membrane specializations also seen in osteoclasts (24, 32). The examination of foamy macrophage and ependymal cathepsin K expression extends our prior studies of matrix proteinase activity from ependymal cells in Axolotl spinal cord regeneration by Chernoff et al. (20).

The studies reported here characterize distribution and functionality of foamy macrophages and some MNGCs in the injury-reactive Axolotl spinal cord meninges, starting at the histological level. In transected adult Axolotl body-region (non-tail) cord, transmission electron microscopic (TEM) morphological studies, plus collagen and proteoglycan staining, show that interstitial meningeal ECM fills the lesion site and wraps the regenerating transected stumps. DiI labeling identify lipid-laden mononucleated cells attached to the fibrotic meningeal ECM. Within the lesion site, lipid-laden and multinucleated cells share ECM-filled space with the reactive ependymal cells that grow out from the spinal cord. Transmission electron microscope (TEM) studies show co-localization of foamy macrophages and reactive ependymal cells within lesion site fibrillar collagen. A primary tissue culture system uses lipid stains to identify neutral and polar lipids. Functional studies indicate that the foamy macrophages and some associated MNGCs take up lipid via the scavenger receptor CD36, co-expressed with toll-like receptor 4 (TLR4), and produce cathepsin K.

Our studies are the first to indicate that foamy macrophages are present in reactive Axolotl spinal cord meninges and participate in spinal cord regeneration.

## Methods and Materials

### Surgery and Tissue Culture

#### Surgery

Axolotls were obtained from the Ambystoma Genetic Stock Center, University of Kentucky and maintained at 20–22°C in 20% Holtfreter's Salts Solution. Transdermal anesthetic tricaine methane sulfonate (Finquel; Syndel, Formerly Western Chemical) was used in 20% Holtfreter's Salts Solution, with thimerosal for disinfection of the surgical field (5 ml/L of a 20 g/L 88% ethanol thimerosal stock), adjusted to pH 7 with sodium bicarbonate. Finquel concentration was adjusted for the size of the animal. Animals were anesthetized in 0.5 g/l Finquel for adult; >20 cm, 2–3 years old; 0.3 g/L for juveniles; 10–15 cm, 6 months old. Before surgery, animals were injected with the antibiotic Amikacin (2.5 mg/ml, 0.75 ml for an adult; 0.5 ml for a juvenile). The lesioning procedure is described in detail in Chernoff et al. (56) and will not be repeated here. Post-surgically, lesioned-cord animals were treated in 20% Holtfreter's solution at 12°C in a BOD incubator for 3 days, with daily water changes and amikacin injections, then kept at 20–22°C in the vivarium through the regeneration process. All husbandry, surgery, analgesia, and euthanasia was performed following the IUPUI School of Science IACUC approved protocol SC 280R.

#### Tissue Culture

**Figure 1:**
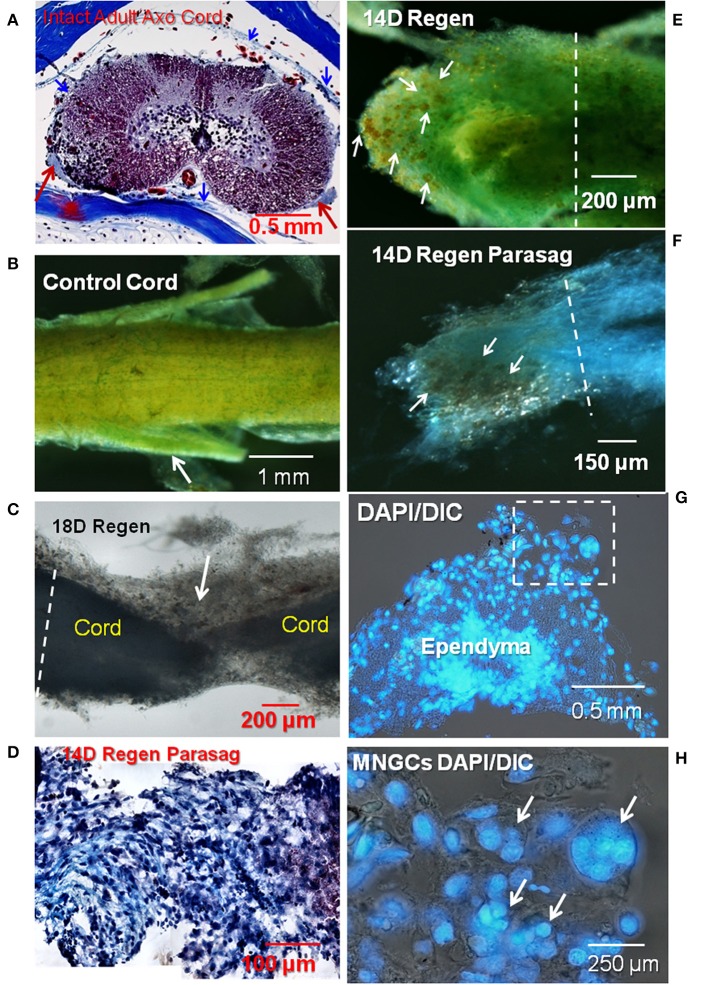
Collagen and proteoglycan stains in regenerating and control adult axolotl spinal cord. **(A)** A cross-section of intact adult axolotl spinal cord labeled with trichrome stain. Red arrows show denticulate ligaments, a pial extension stained with aniline blue (collagen). Thin meningeal layers are present, collagen stained blue (blue arrowheads). **(B)** Spinal cord wholemount labeled with mentanil yellow (collagen) and alcian blue (acidic proteoglycans). Arrow shows a nerve root. **(C)** Unstained lesioned cord regenerated for 18 days, backlit stereoscopic view. Arrow shows a mass of ECM that joins the proximal and distal regenerating stumps. **(D)** Parasagittal paraffin section through 14D regenerating cord stained with trichrome. **(E)** Mentanil yellow and alcian blue label in 14D regenerating cord. The arrows indicate lipid-laden cells that appear orange against the stained ECM. **(F)** Regenerating spinal cord stained with alcian blue and the tissue dissected in the parasagittal plane. Lipid-laden cells in the fibrotic ECM are indicated by arrows. **(G)** Paraffin sections of a regenerating 14D cord with DAPI nuclear label. Stump region with reactive meninges. Dashed square indicates area at higher magnification in **(H)**. **(H)** DAPI-labeled nuclei in 14D regenerating cord. Arrows show a group of multinucleated cells present in the meninges. The white dashed lines in **(C,E,F)** indicate the site margin of harvested tissue for explants culture. Axo, axolotl; Regen, regenerating; Parasag, parasaggital; D, day; DIC, differential interference contrast. Magnification bar is shown in the lower portion of each image.

For these experiments sets of four to six animals, either sex, age matched, but any color morph, were lesioned. Explants were isolated following the procedure described in Chernoff et al. (56) and O'Hara and Chernoff (6). Two weeks outgrowth (see dashed lines in Figure 1) was isolated, cut to size and cultured on poly-D-lysine/fibronectin-coated dishes. Explants were divided among 2–3 dishes. Each experiment was repeated at least three times. Culture contained Leibovitz L-15 medium, 5 mM Hepes Buffer, and progesterone 20 nM. Finally a stock solution containing 5 μg/ml insulin, 100 μg/ml transferrin, 100 μM putrescine, and 30 nM selenium was added (Sigma Chemical). EGF 20 ng/ml, 1% axolotl serum, and 1% Pen-Strep/Fungizone (Gibco) was added to L-15 medium, which was used for the cultures. pH was adjusted to 7.6.

#### Culture Dish Preparation

Thirty-five millimeter polystyrene tissue culture dishes were coated with 100 μg/ml poly- D -lysine in HEPES-buffered saline solution, pH 7.4. The dishes were incubated at 37°C for at least 30 min, then rinsed twice with HEPES buffered saline pH 7.4. Fibronectin (75 μg/ml) was added to each dish and incubated at 37°C for 1 h and rinsed with HEPES buffered saline. HEPES buffered saline contains 0.01 M HEPES, 0.01 M KCl, and 0.013 M NaCl in water, adjusted to pH 7.4. The dishes were rinsed with medium prior to addition of explants.

### Histological Stains

#### Wholemount

Animals were anesthetized and 3 cm spinal column segments were excised fixed in 4% paraformaldehyde, pH 7.4 on a rocker platform at room temperature for 20 min, then overnight at 4°C. After rinsing in Hanks' BSS, the tissue was incubated for 1 day in 0.5% neutral calcium chelator EGTA to soften bone, rinsed in HBSS and the spinal cord removed.

To identify the ECM collagen and proteoglycan wholemount segments of axolotl spinal cord were stained. Proteoglycan was stained with alcian blue alone. Collagen and proteoglycan were co-stained with metanil yellow and alcian blue. Samples were stained in alcian blue for 5 min, and in metanil yellow for 1 min. After staining, samples were rinsed briefly in three changes of 10% ethanol for 5 min to remove excess dye.

All samples were viewed and photographed in their whole-mount state using a Nikon stereomicrocope and photographed using a Nikon DXM-F digital camera system.

#### Trichrome Stain

Slides with paraffin sections were deparaffinized in xylene, twice for 5 min each. The slides were then placed in decreasing concentrations of ethanol, then distilled water for 30 s at each step. Deparaffinized slides were stained using Weigert's iron hematoxylin, followed by Biebrich scarlet-acid fuchin and phosphotungstic/phosphomolybdic acid following kit instructions (Sigma Chemical Co.). Slides were then soaked for 5 min in aniline blue. After the finally staining step, the slides are placed in 1% acetic acid for 2 min, twice, then rinsed in distilled water for 5 min, twice. Finally, slides are dehydrated and cleared in increasing concentrations of ethanol and finally xylene for 10 min in each solution. Histomount (Pella) permanent mounting medium is then used to mount the slides.

### Transmission Electron Microscopy

Animals were anesthetized and either perfused, or spinal cord segments were fixed *in situ* for several minutes in cacodylate-buffered tri-aldehyde fixative (57). Tissue was removed and fixation continued in Kalt's fixative for 24 h. Tissue was post-fixed in buffered 2% osmium tetroxide, dehydrated, and embedded in araldite epoxy resin. Plastic-embedded tissue was sectioned on a Porter-Blum MT-2 ultra-microtome with glass knives for 1-micron sections, or with a diamond knife for thin sections. Thick sections were collected on glass slides and stained with 1% toluidine blue. Thin sections (50–80 nm) were made at selected intervals, collected on bare grids or on Padget-film-supported-1-hole grids, stained with lead-citrate, and examined with a Tecnai G2 12 Bio Twin (FEI, Hillsboro, OR) equipped with AMT CCD Camera (Advanced Microscopy Techniques, Danvers, MA). Two older images were obtained using a Philips 400 transmission electron microscopy.

### Actin Staining

Samples were fixed in 4% paraformaldehyde in HBSS for 20 min. Fixative was rinsed out with multiple changes of Hanks' Balanced Salts Solution (HBSS) pH 7.6. Cultures were permeabilized for 5 min with 0.1% Triton-X-100 in HBSS. Wholemount samples were permeabilized for 10 min. Triton-X-100 was removed with three 5 min distilled water rinses. Rhodamine-Phalloidin (Invitrogen) stock was prepared in methanol as directed, diluted 1:200 into the sample buffer and used at a concentration of 1.5 units/ml. Cultures were incubated for 10 min and wholemount samples were incubated for 20 min, then rinsed in HBSS twice, 10 min each. HBSS was removed and DAPI/antifade (Invitrogen) added to cover the samples in small culture dishes, which were coverslipped and viewed.

### Lipid Labels and Inhibitor

#### DiI Staining

Cell cultures were fixed with 4% paraformaldehyde at room temperature for 20 min, rinsed in HBSS, then incubated with DiI (1 mg/ml in 100% ethanol) for 15 min. Dishes were rinsed briefly in ethanol to remove unbound DiI. Cultures were incubated in HBSS for 1 h to allow DiI to partition from membranes to lipid droplets. Antifade with DAPI (Invitrogen) was added, cultures coverslipped and viewed with a Nikon Eclipse TE 2000-U inverted phase contrast and fluorescence microscope.

#### Oil Red O Staining

For neutral lipid staining, a stock solution of Oil Red O (Sigma Aldrich) was prepared by dissolving 0.25 g/40 ml of 2-propanol. Solution was warmed at 37°C to dissolve the dye. Before use, a 3:2 dilution of the dye/propanol was made with water and 0.22 micron filtered. Working solution was prepared immediately before use. Cultures were fixed in paraformaldehyde, rinsed in HBSS, then water. After a brief rinse with 60% 2-propanol the working dye solution was added for 1–2 min. Staining was observed on an inverted microscope. Dye was removed, dishes briefly rinsed once with 60% 2-propanol then distilled water (10 min) and digital images captured.

#### Polar Lipid Live Stain

To visualize neutral and polar lipids together, the DyRect Live-Cell Neutral Lipid Imaging Kit (Marker Gene Technologies, Inc.) was used. In this live foam cell stain, neutral lipids fluoresce green and polar lipids fluoresce red. The green fluorescence overlaps with green autofluorescence in our cells, so only the polar lipid staining properties were useful. DyRect reagent was reconstituted in ethanol at 1 mg/ml and diluted to 1 μg/ml in culture medium immediately before use. The dye-medium containing cultures were incubated at room temperature for 1 h, rinsed in HBSS and imaged live.

#### Ox-LDL Uptake

DiI-labeled Ox-LDLs (Invitrogen L3482) were diluted to 25 ug/ml in E3 medium (see Axolotl Tissue Culture section for composition) and applied to cultures for 24 h. The cultures were rinsed with HBSS and fresh medium was applied before observation via fluorescence microscopy.

#### CD36 Inhibition

CD36 inhibition medium was created by adding sulfo-N-succinimidyl oleate to E3 medium at a final concentration of 100 uM. Culture dishes were incubated with the CD36 inhibition media for 24 h, then DiI labeled Ox-LDLs (25 ug/ml) were added to the dish for 24 h incubation. Culture dishes were rinsed with HBSS, and fresh E3 media was added before observation under fluorescence microscopy.

### Glucosamine Uptake, Autoradiography

For glucosamine uptake experiments, explant cultures were established and grown for 6 days. Five μCi (185 μBq) of D-[6-^3^H(N)]-glucosamine (Perkin-Elmer) was added per ml of E3 medium and the cultures were incubated for 24 h. Labeled medium was removed and cultures rinsed three times in HEPES buffered saline, fixed in ice-cold 5% acetic acid in ethanol for 10 min at 4°C, rinsed three times with 100% ethanol, and air dried. The dry plates were coated with 0.5 ml Kodak-NB2 nuclear tracking emulsion (Eastman Kodak Co) and allowed to expose for 2 weeks at 4°C in a light-tight box containing desiccant. After 2 weeks the plates were brought to room temperature, developed with Kodak D-19 developer for 10 min at room temperature, rinsed, fixed with Kodafix (diluted 1: 3) for 10 min, rinsed again, and allowed to air dry. Coverslips were applied to the dishes with Histomount permanent mounting medium (Ted Pella).

### Immunohistochemistry

#### Paraffin Sections

Dissected tissues were fixed in 4% paraformaldehyde in HBSS, pH 7.6, at 4°C for at least 1 h, rinsed in HBSS and dehydrated in a graded ethanol series, followed by two xylene rinses, paraffin penetration and paraffin embedding. The sections were cut to 10 μm thickness by microtome and “baked” at 60°C overnight onto Superfrost/Plus Microscope glass slides (Fisher). After deparaffinizing and rinsing with PBT (phosphate-buffered saline, PBS, plus 0.1% Tween-20), the sections were placed in 90°C 0.01 M citrate buffer (pH 6.0) for 10 min, for post-fixation antigen recovery, unless otherwise noted. Sections were then treated with blocking buffer as follows: PBT with 10% normal goat serum (NGS), diluted 1:1 with Superblock (Pierce Chemical). Primary antibody was added in HBSS and incubated overnight at 4°C. After rinsing three times with HBSS, sections were incubated with Alexa Fluor 594 secondary antibody (1:2,000, Invitrogen) for 2 h at room temperature. CD36 antibody (R&D Systems MAB25191) was diluted to 2.5 ug/ml. After washing with HBSS twice, sections were mounted in SlowFade Gold antifade reagent with DAPI as the nuclear counterstain (Invitrogen) and coverslipped. Specimens were observed with a Nikon Eclipse E800 fluorescence/DIC microscope.

#### Cell Cultures

Culture dishes were fixed with 4% paraformaldehyde for 30 min at room temperature and rinsed three times with HBSS before incubation in SuperBlock blocking buffer in PBS (Thermo). Dishes were rinsed three times with HBSS, and the appropriately diluted primary antibody applied and incubated overnight. Then dishes were rinsed three times with HBSS, and Alexa Flour 595 secondary antibody was applied and incubated for 2.5 h, rinsed three times with HBSS, mounted in SlowFade Gold antifade reagent with DAPI and coverslipped. Specimens were observed with a Nikon Eclipse TE 2000-U inverted phase contrast and fluorescence microscope or a Keyence BZ-X Fluorescence, phase, DIC Microscope. TLR4 polyclonal Ab (Novus Bio NB100-56580SS) and TLR4 polyclonal Ab (Novus Bio NB100-56581SS) were diluted to 5 ug/mL in HBSS. CD36 Ab (R&D Systems MAB25191), and CD36 Ab (Novus Bio NB400-145SS) were diluted to 2.5 ug/mL in HBSS. Cathepsin K Ab (Abcam ab19027) was applied at 1:75 dilution in HBSS (antibody information shown in table form in Supplemental Figure 1).

### Myelin Experiments

#### Extraction and Labeling

Twelve axolotl brains were isolated and homogenized in a 0.32 M sucrose solution with a sterile plastic pestle in 1.5 ml microcentrifuge tubes. The homogenate was layered over a 0.8 M sucrose solution and centrifuged at a G-force of 16,000 (Sorvall Biofuge Pico, radius = 8.5 cm, 13,000 rpm) for 15 min. The myelin was collected at the interface of the two sucrose solutions. 1,1′-dioctadecyl-3,3,3′3′-tetramethylindocarbocyanine perchlorate (DiI) in ethanol (1 mg/mL) was applied to the myelin extract and incubated for 30 min at RT. The labeled myelin extract was rinsed three times with HBSS to remove any unbound DiI.

#### Endogeneous Myelin Staining

Fluoromyelin Red (Fisher F34652) was added at 1/300 dilution in HBSS to fixed cultures and incubated for 40 min then rinsed in HBSS. DAPI and antifade (Invitrogen) was added to the dishes and coverslipped. Cultures were imaged with fluorescence microscopy.

#### Myelin Uptake

DiI-labeled myelin extract (100 μL/2 ml culture medium) was added to the 35 mm dish cultures and incubated for 48 h. Dishes were rinsed with HBSS and new E3 media added before cultures were observed by fluorescence microscopy.

### Statistical Analysis

Ninety-two unique photographic fields from >16 cultured explants in untreated controls from seven separate untreated regeneration control experiments were compiled and sorted into categories of outgrowth: condensed ependymal outgrowth with no foam cells, condensed ependymal outgrowth with foam cells, dispersed ependymal cells with foam cells, dispersed ependymal cells without foam cells and mixed forms. Each form was tabulated for each of the seven experiments. Using Graphpad Prism, A one-way analysis of variance (ANOVA) with Tukey's *post-hoc* Multiple Comparisons Test was performed. In this type of analysis significance is first determined by ANOVA, then an stricter adjusted *p*-value is determined for each comparison (58).

## Results

### Overview of Meninges, Meningeal Matrix, and Foamy Macrophages

The results provide a structural overview of the organization of reactive Axolotl meninges, fibrotic ECM and associated foamy macrophages and MNGCs. Control cords were compared with the regeneration stage when invasive meninges have produced interstitial ECM and reactive ependymal cells grow out from the central.

Figure 1A shows a trichrome stained histological cross-section of intact adult lumbar region Axolotl spinal cord. The normal meningeal layers were very thin and stain with aniline blue, indicating the presence of collagen. A stereoscopic image of a wholemount preparation of control cord was stained with metanil yellow for collagen and alcian blue for acidic proteoglycans (Figure 1B). The entire cord and the nerve roots are ensheathed with the collagen-containing meninges (Figure 1B). Small areas of alcian blue represent mainly basal lamina associated with capillaries (Figure 1B). Following transection, meningeal fibrosis produced a mass of interstitial ECM seen here in an unstained stereoscopic wholemount preparation (Figure 1C). The tapered regenerating outgrowth of the cord was embedded in the meningeal matrix. In a parasagittal paraffin section, the trichrome stain showed a large amount of aniline blue-stained fibrillar collagen with cells interspersed (purple) across the lesion site (Figure 1D). The violet material on the right was associated with a fibrin clot.

One bulbous end of a 2-weeks regenerating cord showed several features (Figure 1E). An extensive amount of alcian blue-stained sulfated proteoglycan-containing ECM overlayed metanil yellow-stained fibrillar collagen-containing ECM. This meningeal investment of the regenerating cord stump showed numerous dark orange cells on the ECM (arrows). This appearance reflected the large amount of yellowish lipid seen against the stained ECM. In regenerating spinal cord labeled solely with alcian blue and dissected in a parasagittal plane, the lipid-laden cells were found throughout the fibrotic ECM at the tip of the regenerative outgrowth (Figure 1F).

Organic solvents used to embed material for sectioning extracted most of lipid present in the lipid-laden cells like those shown in Figures 1E,F, precluding lipid staining in paraffin sections. However, DAPI nuclear label in a cross-section of stump near the lesion site showed the presence of numerous multinucleated cells in fibrotic meninges surrounding the spinal cord in the regenerating tissue (Figures 1G,H).

**Figure 2:**
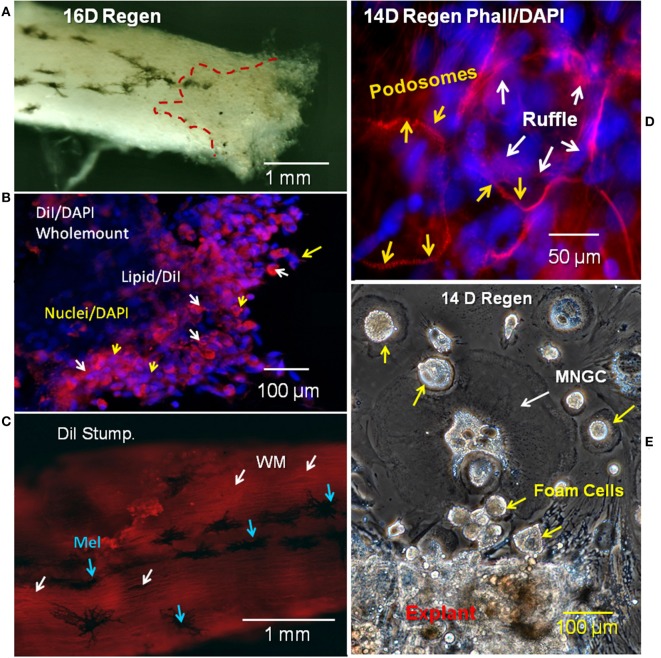
Regenerating cord meninges MNGCs and foam cells. **(A)** Red dashed lines shows the region of regenerating outgrowth with lipid-laden cells in one side of a 16D spinal cord regenerate. **(B)** Lesioned wholemount cord from 16D regenerating cord dual-labeled with DiI (red/lipid) and DAPI (blue/nuclei). Lipid-laden cells are clustered near the regenerating end. White arrows show cells labeled with DiI and yellow arrows show labeled nuclei. **(C)** Image near the surface of 16D wholemount distal stump of a regenerating cord labeled with DiI. White arrows show white matter in axonal tracts, while blue arrows indicate surface melanocytes. **(D)** Regenerating 14D spinal cord wholemount labeled with rhodamine phalloidin (red/actin) and DAPI (blue/nuclei). Podosomes labeled with rhodamine phalloidin (yellow arrows) can be seen surrounding the nuclei of MNGCs. The ruffled border is indicated by white arrows. **(E)** Phase contrast image of unlabeled outgrowth from a 14D regenerate in culture 11 days. MNGCs were detected in the outgrowth (white arrows), in addition to foam cells (yellow arrows). Regen, regenerating; wm, white matter; mel, melanocyte; MNGC, multinucleated giant cells; DIV, days *in vitro*. Magnification bar is shown in the lower portion of each image.

To characterize the lipid-laden cells described in Figure 1, regenerating cord was labeled with the lipophilic membrane stain DiI and the fluorescent F-actin probe rhodamine-phalloidin. Figure 2A shows a wholemount view of unstained spinal cord with outgrowth from one side of a transected, regenerating cord. Dark melanocytes were present on stump meninges, as they are on control cords. The dashed line shows the margin of injury-reactive material accumulated near the cut end (Figure 2A). A region of this regenerating outgrowth is shown in the DiI wholemount stain that follows (Figure 2B). DiI/DAPI labeling showed a region of lipid-containing cells concentrated in the meninges, on the regenerative outgrowth (Figure 2B). These lipid-laden cells were not present on the surface of the stump distant from the transection (~2 cm from the regenerating end; Figure 2C). DiI staining of the distal stump showed only the white matter axonal myelin just beneath the cord surface (Figure 2C).

Rhodamine-phalloidin labeling for F-actin showed a small group of MNGCs on the reactive meninges of another lesioned cord (Figure 2D). The distinctive podosome actin dots of the sealing ring were seen around the margin of the MNGCs, along with regions of the more interior ruffled border (Figure 2D). These structures were localized to the margins of spread MNGCs, like that shown in lesion site outgrowth *in vitro* (Figure 2E). The outgrowth shown in Figure 2E was from a distal end region of the regenerating cord like that shown in Figure 2A.

**Figure 3:**
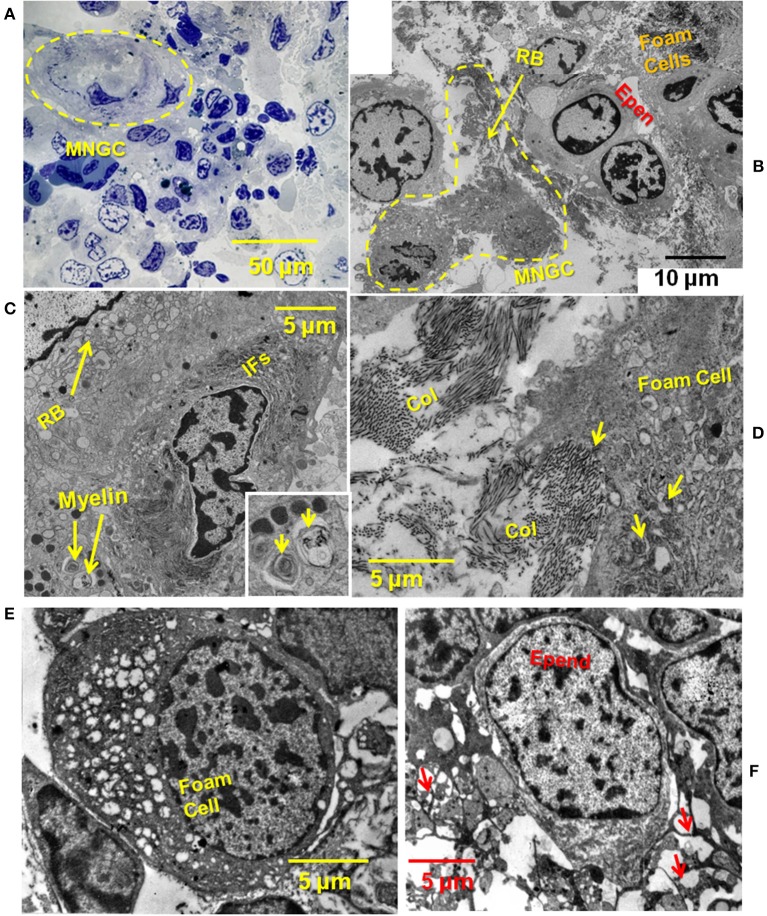
TEM showing close relationship between MNGC, ECM, ependymal cells, and foam cells. **(A)** Thick section of plastic embedded 2-weeks spinal cord regenerate outgrowth stained with toluidine blue. Yellow dashed circle surrounds MNGC. **(B)** Thin section TEM of lesion site shown in **(A)**. A portion of the MNGC is circled in yellow (dashed line). Arrow indicates portion of the ruffled border. Foamy macrophages and ependymal cells are present. **(C)** A portion of the ruffled border, and perinuclear intermediate filaments are shown. Engulfed myelin fragments are present within the cytoplasm. Engulfed myelin enlarged in inset (arrows) **(D)** Foamy macrophage engulfing fibrillar collagen within the lesion site. Arrows show intracytoplasmic collagen. **(E)** Deeper within the lesion site a foamy macrophage is present among ependymal and meningeal cells. **(F)** Deeper within the lesion site an ependymal cells show a network of processes (arrows). MNGC, multinucleated giant cells; RB, ruffled border; RBC, red blood cell; Epen, ependymal cells; Ifs, intermediate filaments; Col, collagen; Mening, meningeal cell; macr, macrophage; Nucl, nucleus. Magnification bar is shown in the lower portion of each image.

Fine-structural detail of ECM, ependymal cells, foam cells and MNGCs in regenerating Axolotl cord was obtained by transmission electron microscopy (TEM). A toluidine blue-stained plastic thick section of a 2-weeks lesion site showed an overview of a lesion site examined by TEM (Figure 3A). A MNGC, in which nuclei were visualized through several sections, is circled with a dashed yellow line. TEM examination of this area showed juxtaposition of the MNGC, ependymal cells and foam cells (Figure 3B, enlarged area Supplemental Figure 2). At increased magnification, masses of vimentin intermediate filaments were seen around one of the MNGC nuclei [Figure 3C; (59)]. An enlargement of the zone of intermediate filaments is shown in Supplemental Figure 2B. The dark structure next to the MNGC appeared to be a telopode [Figure 3C; (60)].

Figure 3D shows a higher magnification view of an area between foam cells and ependymal cells. A large amount of fibrillar collagen was present and appeared to be in the process of engulfment (Figure 3D). Figures 3E,F shows a foam cell among meningeal cells, deeper in the lesion site. Fibrillar collagen was absent from this zone. Other white blood cells are found within the 2 weeks regenerate lesion site: Supplemental Figures 3A,B show a macrophage engulfing red blood cells and a lymphocyte among ependymal and meningeal cells (61, 62).

**Figure 4:**
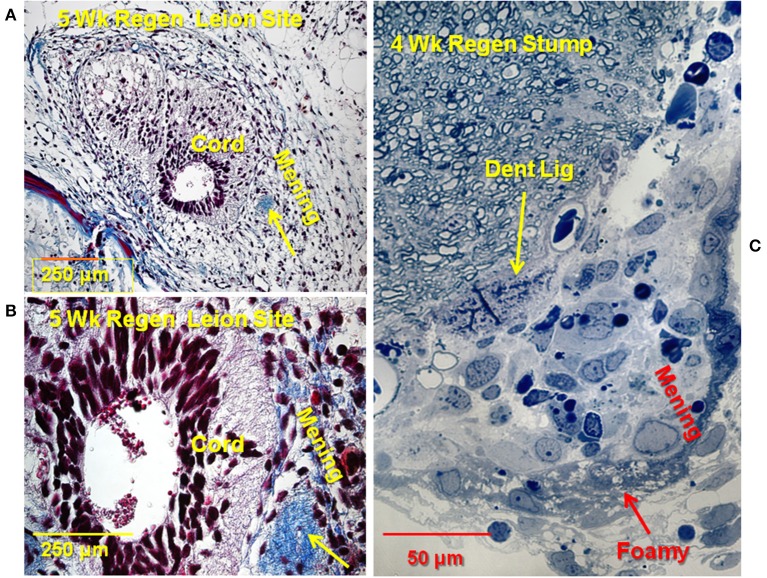
Later stage of the lesion site: rejoined cord. **(A)** Trichrome stained section through 5 weeks regenerate lesion site. Cord is asymmetrical, with enlarged central canal, white matter not fully reconstructed. Extensive reactive meninges around the cord. Arrow: denticulate ligament. Collagen is blue. **(B)** Higher magnification view of a trichrome stained section through the 5 weeks regenerate. Extruded material visible in central canal Arrow: denticulate ligament. **(C)** Toluidine blue stained plastic thick section 4 weeks regenerate. Stump near lesion site. Reactive meninges with white blood cells and some foamy cells (red arrow), area of denticulate ligament (yellow arrow). Magnification bar shown in the lower portion of each image. Dent Lig, denticulate ligament; Mening, meninges; Wk, week.

Later in the regeneration process ependymal outgrowth re-epithelializes to reform the central canal, re-extend radial processes, re-form endfeet and form channels through which regenerating axons extend (2, 5, 10). Figure 4 shows the 4–5 weeks period of regeneration at which the regenerating ends of the cord had met and axonal regrowth is underway to restore the full thickness spinal cord. In Figure 4A, a trichrome-stained paraffin cross-section of a 5 weeks regenerate lesion site shows an asymmetrical regenerate with enlarged central canal. The meninges were excluded to the periphery and were still highly reactive, filling the entire space between the regenerating cord and neural arch and vertebral body. Figure 4B shows a higher magnification view of a section like Figure 4A. The enlarged central canal contained extruded cellular material. The collagenous denticulate ligament (arrow) was adjacent to the regenerating cord. This is significant because the denticulate ligament is an extension of the *pia mater*, and indicated pial restoration around the regenerating cord. A toluidine-stained plastic section showing a cross-section of a proximal stump from a 4 weeks regenerating cord showed the reactive meninges adjacent to the white matter (Figure 4C). This region contained a denticulate ligament profile, comparable to Figures 4A,B. The reactive meninges contained foamy cells and a variety of white blood cells and meningeal cells (Figure 4C).

### Ependymal and Foamy Macrophage Lipid Content and Uptake

The presence of neutral and polar lipid in both ependymal cells and foamy macrophages was shown by using Oil Red O stain for neutral lipid, and DiI staining and DyRect, a proprietary stain, for polar lipids. CD36 was identified as a significant lipid transporter in these cells, in association with TLR4, through antibody localization *in vivo* and *in vitro* and treatment with a CD36 inhibitor *in vitro*. Exposure to DiI-labeled Ox-LDL showed the capacity for additional uptake of Ox-LDL *in vitro* in foamy macrophages.

### Neutral and Polar Lipids

**Figure 5:**
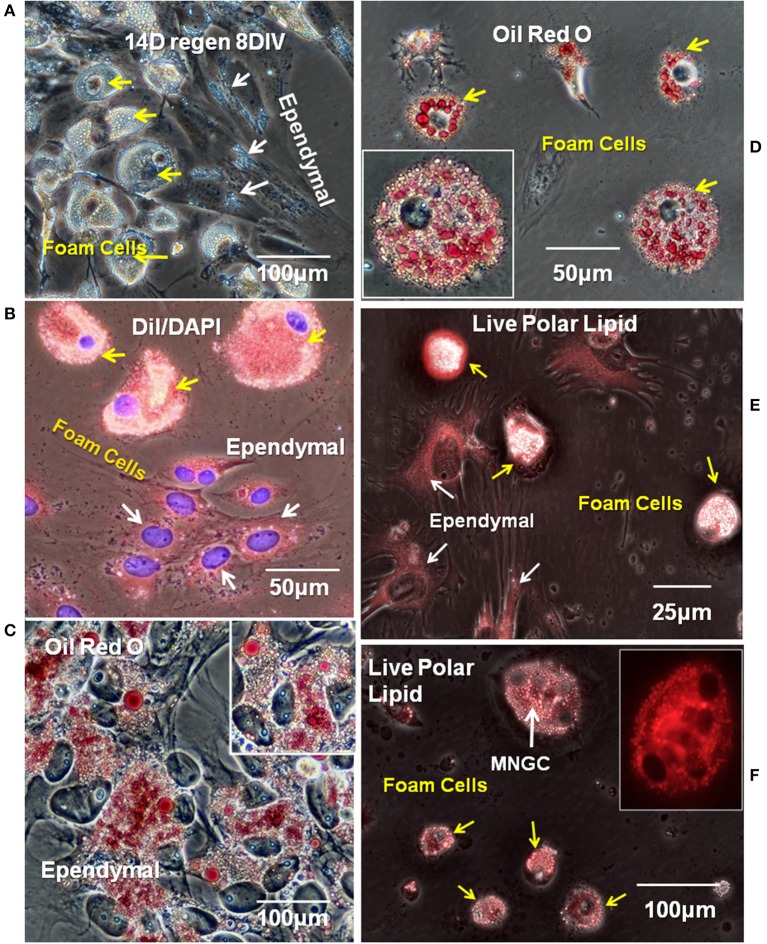
Ependymal cells and foamy macrophages take up neutral and polarized lipids in regenerating spinal cord explants. **(A)** Lipid droplets were apparent ependymal (white arrows) and foamy macrophages (yellow arrow) in unstained explant cultures of 14 days regenerating spinal cord. Phase contrast image **(B)** Ependymal (white arrows) and foamy macrophages (yellow arrows) in regenerating explant cultures are labeled with polar lipid marker DiI. Fluorescence/Phase contrast image. **(C)** Ependymal cell lipid droplets label with neutral lipid marker Oil Red O. Phase contrast image. **(D)** Foamy macrophages lipid droplets also label with neutral lipid marker Oil Red O. Phase contrast image Insets in **(C,D)** show higher magnification images of ependymal cells and foamy cells, respectively. **(E,F)** Ependymal cells (white arrows in **E**), foamy macrophages (yellow arrows in **E,F**), and MNGCs (white arrow in **F**) all labeled with a commercial polar lipid marker. Fluorescence/Phase image, Inset in **(F)** shows a higher magnification of an MNGC with 4 nuclei, fluorescence only. Regen, regenerating; DIV, days *in vitro*. Magnification bar is shown in the lower portion of each image.

When 14D regenerating spinal cord outgrowth was placed in culture, accumulation of small lipid droplets in phase contrast images of live ependymal outgrowth, and a larger mass of lipid in foamy macrophages can be clearly seen (Figure 5A). The fluorescent lipophilic cationic indocarbocyanine dye, DiI, is used here as a direct lipid probe. The amount of DiI stained foamy macrophage lipid was greater, and lipid droplets generally larger, than that in ependymal cells, but both cell types were labeled (Figure 5B). To examine the presence of neutral lipids, Oil Red O staining was performed. Oil Red O stains both ependymal cells and foamy macrophages showing that some of the lipid in both cell types was neutral lipid (Figures 5C,D). At higher magnification both labeled and unlabeled lipid droplets can be seen in foamy macrophages and ependymal cells (Figures 5C,D insets).

Identification of the types of lipid labeled by DiI has never been clear, beyond its known intercalation behavior into cell membrane phospholipid layers (63). While this implies an affinity for polar lipids, the targets are not certain. To further explore polar lipid content, a proprietary polar lipid label was applied to live cultures. Ependymal and foam cells both showed polar lipid content (DyRect Kit; Figures 5E,F). The polar lipid label showed strong overlap with the DiI staining, suggesting polar lipid components were detected by DiI in our cells. In addition to mononucleated foamy macrophages, small MNGCs with lipid also stained strongly for polar lipids (Figure 5F). The Figure 5F inset indicates the location of 4 nuclei. Nuclear counterstaining was not possible because this was a live cell labeling process and cells could not be permeabilized. The neutral lipid staining properties of the DyRect reagent were not useable in the Axolotl cells. The neutral lipid probe fluorescence overlapped too strongly with green autofluorescence and was of no utility (see Supplemental Figure 4).

### Lipid Transporters

The fatty acid translocase/lipid scavenger receptor CD36 is the best candidate for lipid transport in the injured Axolotl spinal cord based on foamy macrophage behavior in other tissues (25). An antibody was identified that reacted with CD36 in paraffin-embedded Axolotl tissue and *in vitro*. In other sources of foam cells, TLR4 co-localizes with CD36 and assists in the lipid transport process, so TLR4 localization was also examined (40). A combination of two TLR4 antibodies to different TLR4 sites was identified that reacts with strongly Axolotl cells *in vitro*, and was effective in paraffin sections.

**Figure 6:**
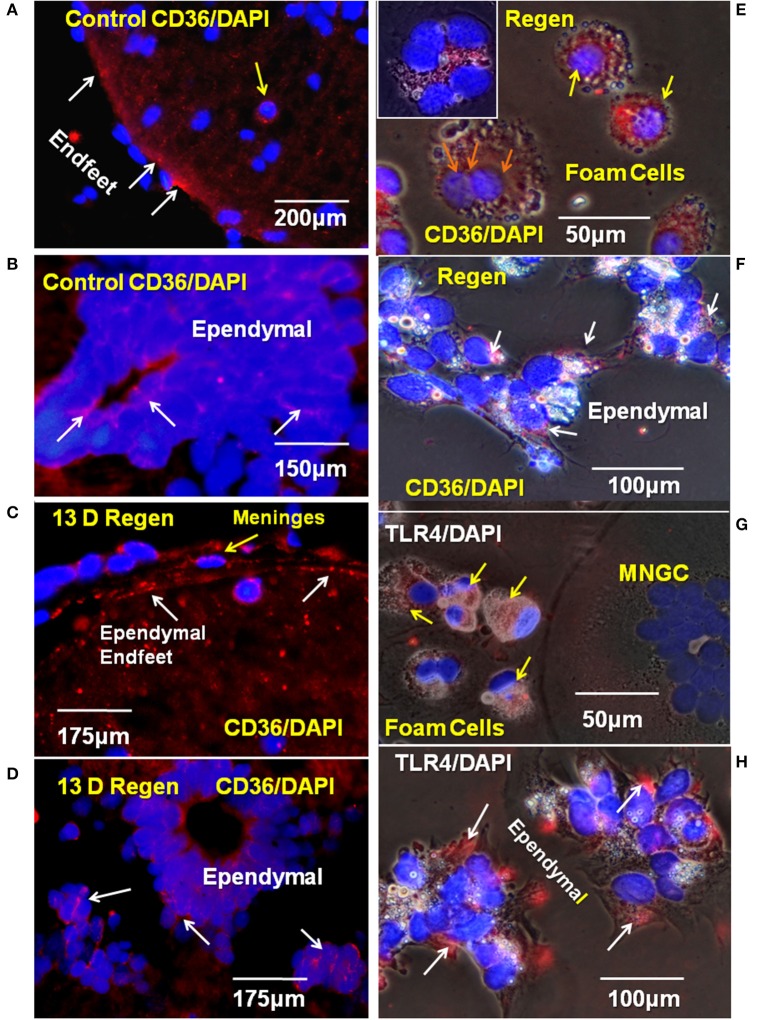
CD36 and TLR4 in regenerating cord *in vivo* and *in vitro*. **(A)** The scavenger receptor CD36 was detected on the endfeet of ependymal cells in control cord paraffin cross-sections. Fluorescence image. **(B)** CD 36 is also present on ependymal cell bodies in intact adult Axolotl spinal cord. Fluorescence image. **(C)** In paraffin sections from regenerating cord stump CD36 was detected in ependymal endfeet in the reactive meninges. Fluorescence image. **(D)** CD36 also present on ependymal cell bodies in regenerating cord, proximal stump. Fluorescence image. **(E)** *In vitro*, CD36 is found on foamy macrophages from 10D regenerate spinal cords 6 days *in vitro* (yellow arrows). Orange arrows show three nuclei in CD36^+^ MNGC. Inset in **(E)** shows a CD36^+^ MNGC with six nuclei. **(F)** CD36^+^ Ependymal cells (white arrows) in explant cultures from a 10D regenerate spinal cord 6 days *in vitro*. **(G)** TLR4 was detected in foamy macrophages (yellow arrows) in culture on cells from 14 days regenenerates, 17 days *in vitro*. **(H)** Ependymal cells from 14 days regenenerates, 17 days *in vitro* are also TLR4^+^ (white arrows). D, day; Regen, regenerating; MNGC, multinucleated giant cells; TLR4, toll like receptor 4. Magnification bar is shown in the lower portion of each image.

In intact adult Axolotl spinal cord sections, the lipid scavenger receptor CD36 was seen in the ependymal endfeet and in small zones of the ependymal cell bodies (Figures 6A,B). In the proximal stump region of the lesioned spinal cord, CD36 was expressed in the reactive meninges, as well as the ependymal cells (Figures 6C,D). *In vitro*, CD36 was strongly expressed by foamy macrophages and small MNGCs (Figures 6E inset). Reactive ependymal cells retained CD36 expression *in vitro*, as well (Figure 6F). Localization of CD36 in foamy macrophages was, generally, central/perinuclear and overlapped the zone of lipid droplets (Figure 6E). CD36 was also present in ependymal cells (Figure 6F).

TLR4 was expressed in both the foamy macrophages and ependymal cells (Figures 6G,H). The region of TLR4 localization in ependymal cells was comparable that seen for CD36 expression, but smaller that that seen in the foamy macrophages (Figures 6F,H). TLR4 was not detected in large, lipid droplet-free MNGCs (Figure 6G).

### Ox-LDL Uptake *in vitro*

**Figure 7:**
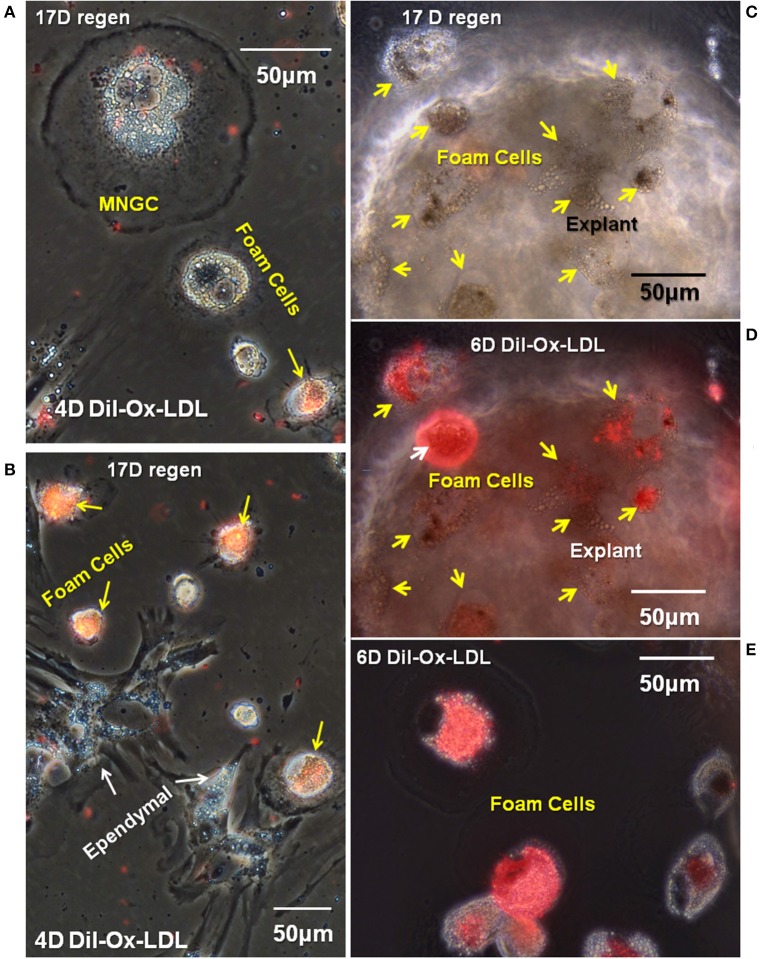
A subpopulation of foamy macrophages takes up DiI-labeled oxidized low-density lipoprotein receptor. Seventeen days cord regenerates were cultured for 11 days *in vitro* **(A,B)** or 17 days *in vitro* **(C–E)** and were treated with DiI-Ox-LDL for 4 days **(A,B)** or 6 days *in vitro* **(C–E)**. MNGCs and ependymal cells (white arrows) were not labeled; however, a subpopulation of foamy macrophages (yellow arrows) were labeled. **(C,D)** Are the same field, with the explant shown in phase contrast in **(C)** and overlaid with fluorescence to highlight the foamy macrophages that have taken up DiI-Ox-LDL (yellow arrows). **(E)** Shows foamy macrophages that have migrated out of the explants and taken up DiI-Ox-LDL in culture. D, day; Regen, regeneration. Magnification bar is shown in the upper portion of images A and E, and the lower portion of images **(B–D)**.

At the time of isolation, all lesion site foamy macrophages and ependymal cells contained lipid acquired *in situ* and both cell types expressed lipid transporters (Figures 5, 6). Results shown in Figure 7 address the ability of foamy macrophages and reactive ependymal cells to take up additional lipid *in vitro*. Explant cultures were established and exposed to DiI- Ox-LDL. A subset of foamy macrophages took up significant amounts of DiI-Ox-LDL (Figures 7A–E). High uptake foam cells were found migrating on the plastic dish, as well as on and under the explants (Figures 7A,B,D,E). The limited number of MNGCs seen in these cultures did not take up DiI-Ox-LDL (e.g., Figure 7A). Ependymal cells also did not take up DiI-Ox-LDL *in vitro* (Figure 7B). All of the foamy macrophages and ependymal cells contained extensive lipid stores (Figures 7A–C), so it is not clear whether those not actively transporting did so because they had reached maximum LDL content, or whether the culture conditions were not fully optimal for lipid uptake.

**Figure 8:**
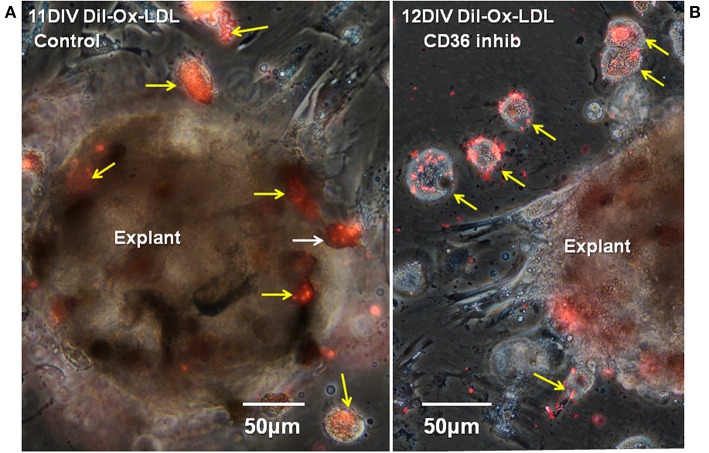
CD36 inhibition reduces DiI-Ox-LDL uptake by foamy macrophages. Seventeen days regenerate spinal cord regenerate explants, were treated with DiI-Ox-LDL at 10DIV for 24 h as the control **(A)** or with the CD36 inhibitor sulfo-N-succinimidyl oleate added at 10DIV plus DiI-Ox-LDL added at 11DIV for 24 h. **(B)** Inhibitor-treated explants showed less uptake of DiI-Ox-LDL in comparison to control. D, day; DIV, days *in vitro*; Ox-LDL, oxidized low-density receptor; inhib, inhibitor; Regen, regeneration. Magnification bar is shown in the lower portion of each image.

**Figure 9:**
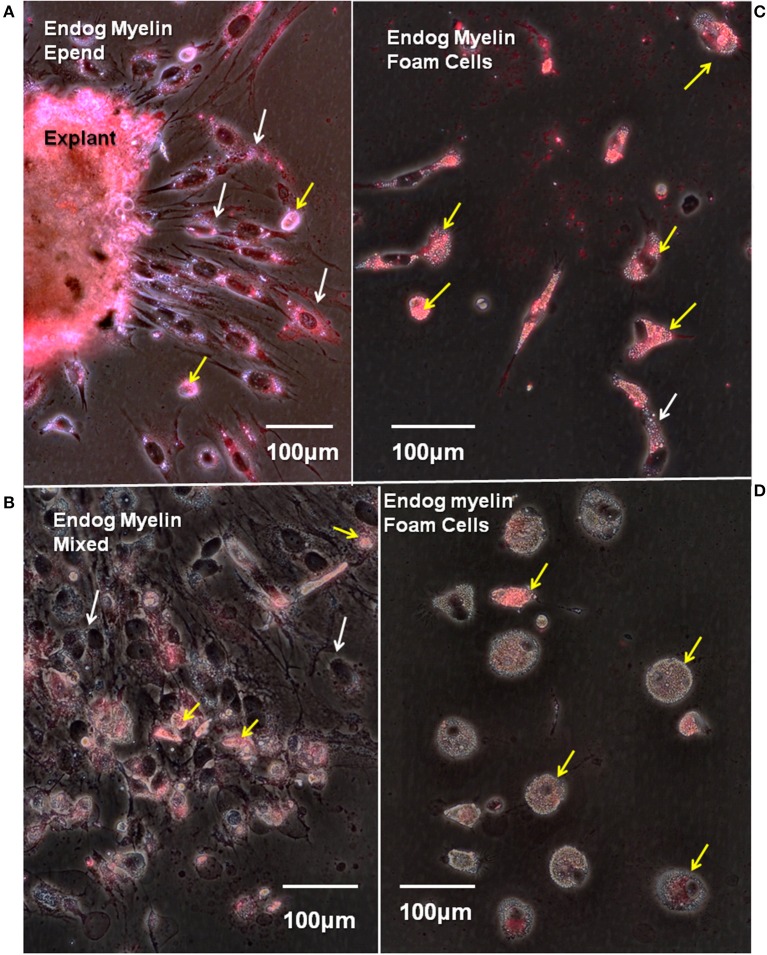
Endogenous myelin uptake is robust in ependymal cells and foamy macrophages. Fourteen days regenerate spinal cord explants were cultured for 8 days *in vitro* and stained with fluoromyelin label to reveal endogenous myelin uptake from the lesion site. Fluorescence/phase images. **(A)** The explant periphery is heavily labeled as is a zone of ependymal outgrowth (white arrows). There are a few, scattered myelin-containing foam cells (yellow arrows). **(B)** In a mixed zone of outgrowth foam cells are labeled (yellow arrows), but ependymal cells show little label (white arrows). **(C)** A zone with heavily myelin-laden foam cells (yellow arrows). White arrow shows one myelin-laden ependymal cell. **(D)** A zone of foam cells with little endogenous myelin uptake (yellow arrows). Endog, endogenous; Epend, ependymal. Magnification bar is shown in the lower portion of each image. Magnification bars are in the lower portion of images.

Functional involvement of CD36 in the *in vitro* Ox-LDL uptake was shown by combining DiI-Ox-LDL uptake with treatment using the CD36 inhibitor sulfo-N-succinimidyl oleate, a modified fatty acid (64). Typical dense DiI-Ox-LDL uptake label was seen in actively transporting control cultures (Figure 8A). Co-treatment with the Ox-LDL label and the oleate inhibitor reduced the area of uptake to small patches and streaks (Figure 9B). This inhibition of Ox-LDL uptake shows a functional role for CD36 in lipid uptake in the Axolotl spinal meninges foam cells.

### Myelin Uptake

Myelin fragments produced from damaged white matter in urodele SCI are seen within lesion site cells in TEM studies [Figure 2C; (5, 12)]. To determine whether the foamy macrophages and/or ependymal cells were serving this function in our experimental system, the presence of myelin engulfed *in situ* (lesion site myelin uptake) was examined along with the ability of lesion site cells to take up additional myelin *in vitro*.

To avoid the enormous amount of autofluorescence found in Axolotl lesion site white matter, endogenous myelin content was assayed by staining primary cultures with fluoromyelin red. Fluoromyelin-labeling of myelin fragments taken up while *in situ*, were seen in cells within the lesion site explants (Figure 9A), in ependymal cells (Figures 9A,B) and foamy macrophages migrating on the culture dishes (Figures 9B–D). In areas of outgrowth showing large numbers of foam cells mixed with ependymal cells, more myelin label appeared in the foam cells (Figure 9B). Within a given culture there were areas of foam cells with different amounts of myelin stain (Figures 9C,D).

**Figure 10:**
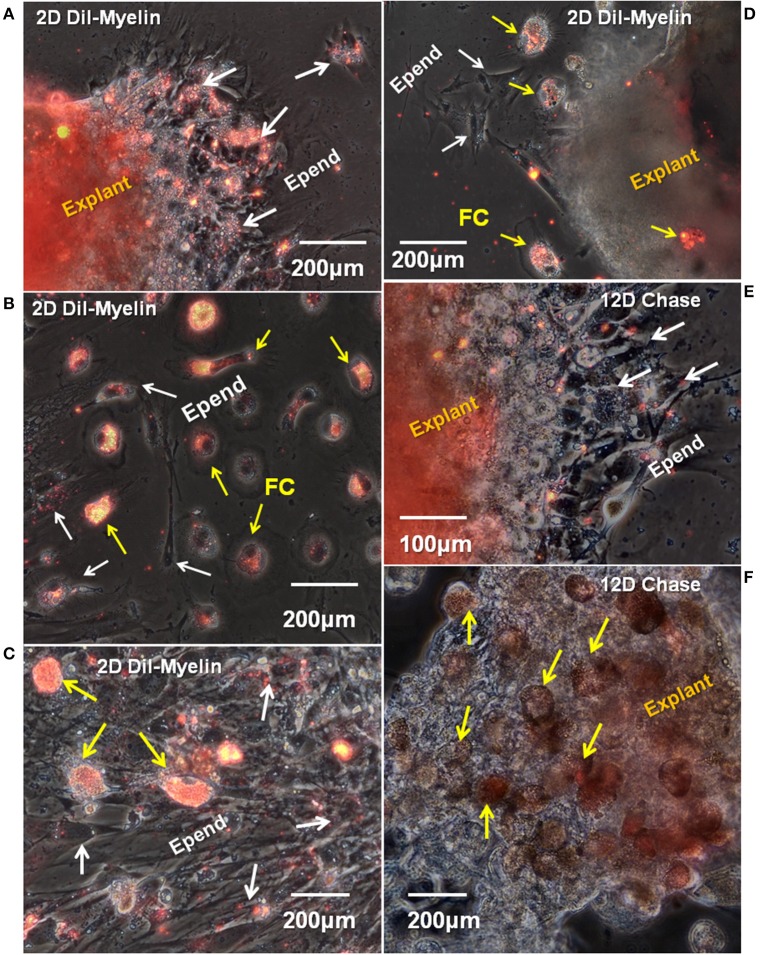
*In vitro* uptake and turnover of myelin in ependymal cells and foamy macrophages. Fourteen days regenerating cord explants were cultured for 8 days, then incubated with DiI-labeled Axolotl brain myelin for an additional 2 days. A representative combination of outgrowth and explants labeling images are shown. **(A)** In a region with ependymal cell outgrowth only, the explants and ependymal outgrowth took up significal levels of labeled myelin fragments (white arrows). **(B)** In an area of mixed, dispersed foam cell and ependymal cell outgrowth, only the foam cells are heavily labeled. **(C)** An denser area of dispersing ependymal cells (white arrows) plus foam cells (yellow arrows) shows heavy myelin uptake *in vitro* only in the foam cells. **(D)** A sparsely labeled explants has strongly labeled foam cell outgrowth (yellow arrows) and unlabeled ependymal cells (white arrows). **(E,F)** Show a 12 days myelin-free chase period following 2 days of DiI-Axolotl-myelin uptake. **(E)** Shows part of the same region shown in **(A)**. Labeled myelin in the ependymal outgrowth is greatly reduced (white arrows). The explants is still labeled. **(F)** Even after the 12 days chase period, foam cells. D, day; Epend, ependymal; FC, foam cell. Magnification bar is shown in the lower portion of each image.

The continuing ability of lesion site cells to take up myelin fragments was also assayed *in vitro*. The probe was DiI-labeled Axolotl brain myelin. New myelin uptake was seen by cells within the explants (Figures 10A,E,F). In the cellular outgrowth, some zones of ependymal cells showed extensive myelin uptake (Figure 10A), while in others there was little (Figures 10C,D). The regions with extensive ependymal myelin uptake appeared to be free of foam cells, as in Figure 10A. Foamy macrophages showed robust DiI-myelin uptake *in vitro* (Figures 10B–D). A 2-days period of exposure was used for the labeled myelin uptake. Cells were cultured without DiI-myelin for an additional 12 days, during which the ependymal cells appeared to turn over most of the labeled myelin (Figure 10A compared with Figure 10E). During the 12 days chase period foamy macrophages seemed to retain more of the labeled myelin, or turnover had released free DiI in these cells which partitioned into lipids stored within the cells (Figure 10F).

### Cathepsin K

**Figure 11:**
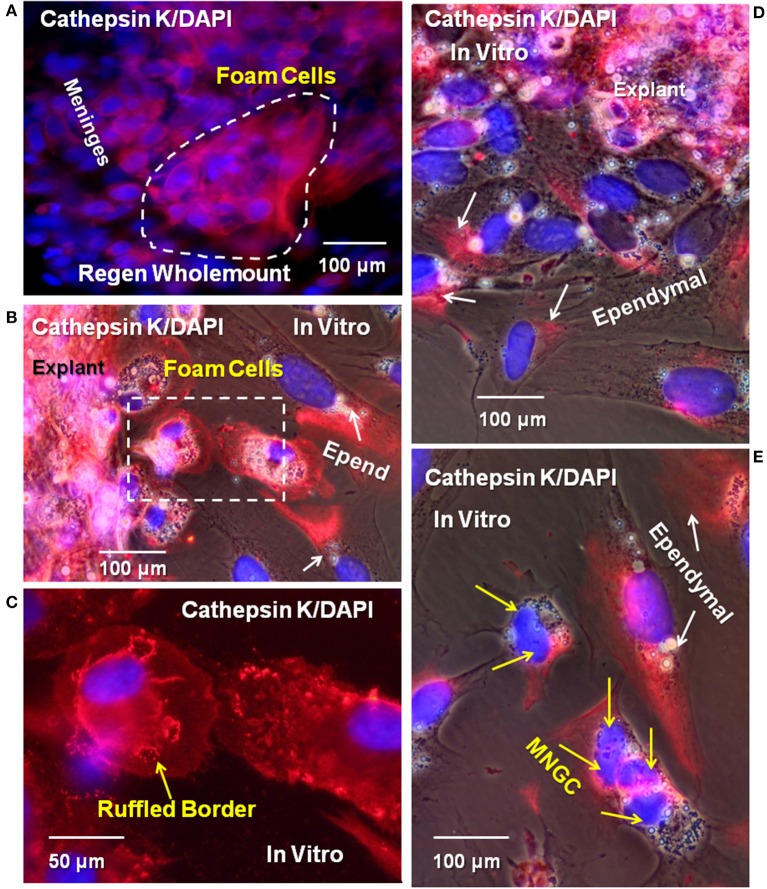
Cysteine protease cathepsin K was detected in meninges, ependymal cells, foamy macrophages, and MNGCs *in vitro* and *in vivo*. **(A)** Wholemount cord tissue from 16D regenerating cord were co-labeled for cathepsin K (red) and DAPI (blue). Cells on the surface of the meninges are positive for cathepsin K (dashed white line). Explants from 16D regenerate cord, cultured for 10DIV, showed labeled ependymal cells **(B,D,E)**, foamy macrophages (**B**, dashed square), and an MNGC **(E)**. Yellow arrows indicate foam cells, white arrows indicate ependymal cells. Ruffled borders sequestering cathepsin K between foam cells and the culture substratum were also detected (yellow arrow, **C**). Yellow arrows indicate nuclei in MNGCs positive for cathepsin K in **(E)**. Regen, regenerating; D, day; DIV, days *in vitro*; MNGC, multinucleated giant cells. Magnification bar is shown in the lower portion of the images.

The cysteine protease cathepsin K catabolizes several ECM molecules, including collagen, elastin, and gelatin. We hypothesized that cathepsin K present in Axolotl cells within the regenerating cord might be involved in catabolizing the fibrous collagen in the lesion site, allowing the regenerating cells to form a more regeneration-friendly region in which new ECM might be secreted. To determine if cathepsin K was expressed in the lesion site, cells *in vivo* and *in vitro* were labeled with cathepsin K antibody. Figure 11A shows a reactive meningeal flatmount from the lesion site where cells were labeled with cathepsin K antibody. In culture, there was expression of the enzyme in cells within the explants and in foamy macrophages and ependymal cells growing out of the explants (Figures 11B–E). In favorably oriented foamy macrophages, the cathepsin K was seen concentrated between the foam cell and the plastic substratum within the sealing ring (Figures 11B,C). In migrating ependymal cells, substantial portions of the cytoplasm were cathepsin K-positive (Figures 11D,E). Small MNGCs also expressed cathepsin K (Figure 11E).

### Glycosaminoglycan Synthesis

Both foam cells and ependymal cell produce ECM-degrading enzymes [Figure 11; (20)], but ependymal cells also rebuild the regenerating spinal cord, including reforming the basal lamina of the *glia limitans* (10). Comparison of the ECM synthetic capacity of foamy macrophages and ependymal cells was performed using ^3^H-glucosamine incorporation and autoradiography as an assessment of glycosaminoglycan and proteoglycan synthetic capacity (65).

**Figure 12:**
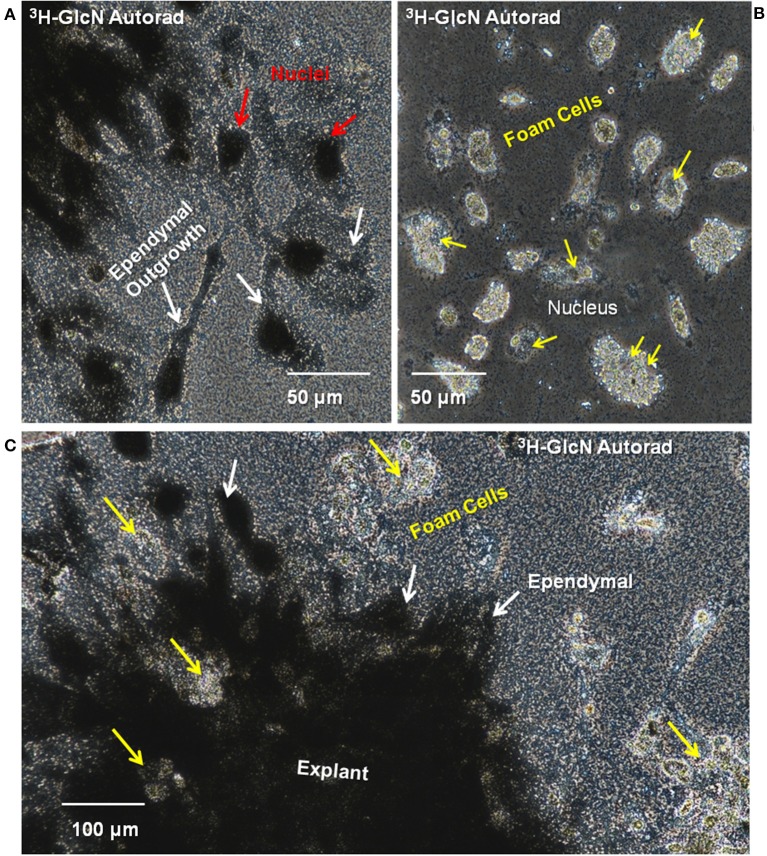
Ependymal cells in cord explant cultures synthesize ECM components. Synthesis of glycosaminoglycan and proteoglycan was investigated by incubating explants with ^3^H-glucosamine and performing culture dish autoradiography. **(A)** Silver grains were detected in the ependymal cell nuclei (red arrows) and cytoplasm (yellow arrows). **(B)** No silver grains were deposited on or around the foamy macrophages. Yellow arrows indicate foamy macrophage nuclei. **(C)** Lesion site explants is heavily labeled with silver grains. Patches of unlabeled foamy macrophages are found within the explants (yellow arrows) and beyond the ependymal cells (white arrows). 3H, tritiated; GlcN, glucosamine; Autorad, autoradiography. Magnification bars are shown in the lower portion of the images.

Extensive ^3^H-glucosamine uptake by mesenchymal ependymal cells occurred over a 24-h incubation in established cultures (Figures 12A,C). Label occurs in the cytoplasm and nucleus, and there is deposition of material onto the substrate near the explants where ependymal cells were most densely distributed. The nuclear label is associated with nuclear pore complex incorporation of O-linked N-acetyl glucosamine synthesized from the ^3^H-glucosamine (66). No incorporation of ^3^H-glucosamine was seen in or around any of the foamy macrophages, identified on the basis of their lipid droplet content comparable to those cells labeled with Oil Red O and DiI (Figures 5, 12B,C). This indicates a major difference in lesion-site remodeling roles between ependymal cells and foamy macrophages.

### Foam Cell/Ependymal Interaction

**Figure 13:**
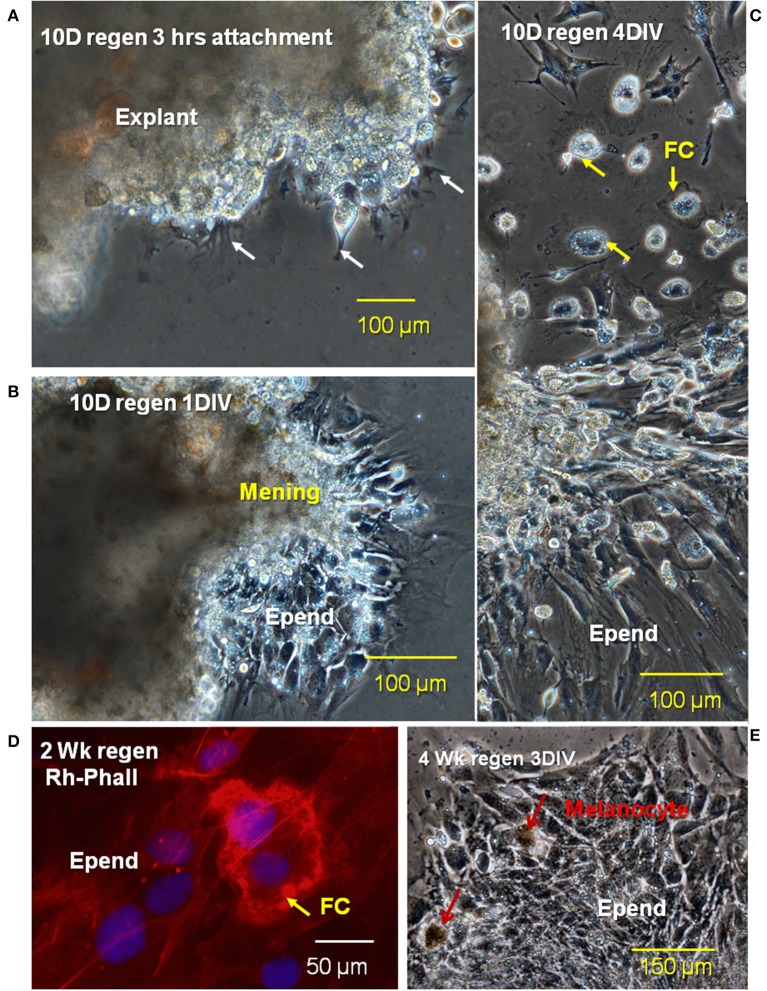
Stages of migration of ependymal and foam cells from lesion site explants *in vitro*. Phase images of a 10D regenerating cord explant showing three different stages of cellular outgrowth; **(A)** at 3 h after the beginning of culture, the explant was firmly attached to the fibronectin-coated dishes and cellular processes begin to extend away from the edges of the explant **(B)**, after 1D *in vitro* (1DIV), ependymal cells are migrating out of explants, while meningeal tissue is spreading, **(C)** after 4DIV, there is extensive dispersed ependymal outgrowth as well as foamy macrophages on and among the ependymal cells **(D)**. Rhodamine-phalloidin label of a cultured 2 weeks regenerating cord (red) showed the sealing ring of a foamy macrophage (FC arrow). **(E)** Cultured 4 weeks regenerates showed no foamy cells attached to ependymal cells. Regen, regenerating; hrs, hours; D, day; DIV, days *in vitro*; mening, meningeal cells; Epend, ependymal cells; Rh-Phall, rhodamine-phalloidin. Magnification bar is shown in the lower portion of each image.

To observe foam cell interaction with the ependymal cells, regenerating tissue was placed in culture conditions that maintain mesenchymal ependymal outgrowth and proliferation of reactive Axolotl spinal cord ependymal cells (56). Pieces of tissue with reactive meninges and ependymal cells grown in culture typically showed three stages of growth: (1) at 3 h after the start of culture, the attachment period, the explant was firmly adherent and cells at the margins started to extend processes (Figure 13A), (2) after 1 day of culture (Figure 6B), injury-reactive ependymal cells were migrating out of the explants and meningeal tissue was spreading, and (3) at 4 days in culture, the explants had extensive, dispersed ependymal outgrowth with foamy macrophages on and among the ependymal cells (Figure 13C). The extensive outgrowth period persists though at least 22 days *in vitro*.

Labeling of cultured 2-weeks regenerating spinal cord with fluorescent-phalloidin showed localization of F-actin in sealing rings of foamy macrophages on the ependymal outgrowth (Figure 13D). At 4 weeks of regeneration, the ependymal outgrowth was reconnected between the cranial and caudal stumps (10). When 4-weeks lesion site regenerate tissue was excised and cultured, the foam cells were no longer localized with the ependymal cells (Figure 13E).

**Figure 14:**
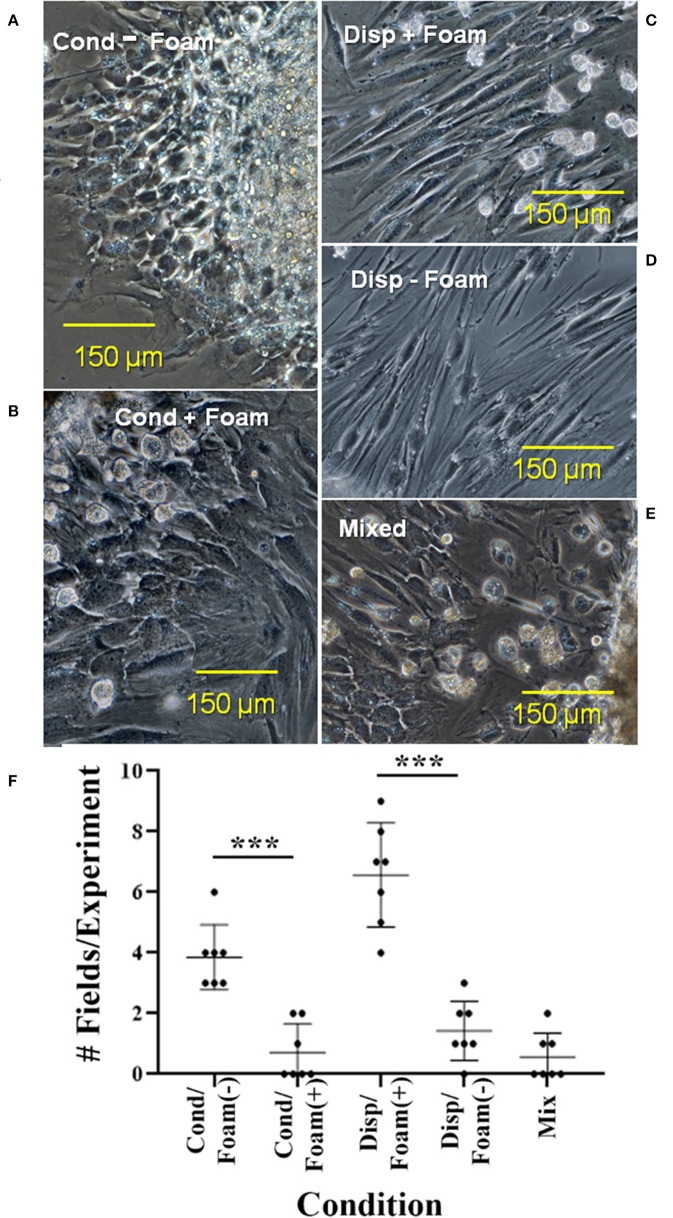
Five types of cellular interactions between ependymal cells and foam cells in cultured regenerative outgrowth. Cultures in which foam cells were associated with condensed or dispersed ependymal cells from 14 to 17D spinal cord outgrowths were quantitated. Dispersed ependymal cells show a preference for associating with foam cells: **(C)** As opposed to condensed ependymal cells **(A,B)** or mixed condensed and dispersed ependymal cells **(E)**. **(D)** Shows a limited region of dispersed ependymal cells without foam cells. **(F)** Shows a graph depicting the quantitation of cultures. Cond, condensed; disp, dispersed. ***p ≤ 0.0001. Magnification bar is shown in the lower portion of each image.

In analysis of cellular arrangement in culture, outgrowth could be divided into 5 forms of foamy macrophage/ependymal interaction: (1) condensed outgrowth with no foamy macrophages (Figure 14A), (2) condensed outgrowth with foamy macrophages (Figure 14B), (3) dispersed outgrowth with foamy macrophages (Figure 14C), (4) dispersed outgrowth without foamy macrophages (Figure 14D), and (5) mixed (Figure 14E). Ninety-two photographic fields from >16 explants in 7 experiments were sorted into the categories listed above. A one-way analysis of variance (ANOVA) with a *post-hoc* Tukey analysis showed a highly statistically significant difference between the presence of foamy cells with dispersed vs. condensed ependymal cells (Figure 14F). The occurrence of dispersed ependymal growth with foamy macrophages was far more prevalent when compared to the dispersed ependymal cells without foamy macrophages. This difference is highly significant with a *p*-value < 0.0001. Conversely, a condensed form of reactive ependymal cell outgrowth occurs in the absence of foam cells, compared to the occurrence of condensed outgrowth in the presence of foam cells (*p*-value < 0.0001). Further test details are included in Supplemental Figure 5.

## Discussion

### Diagrammatic Summary

**Figure 15:**
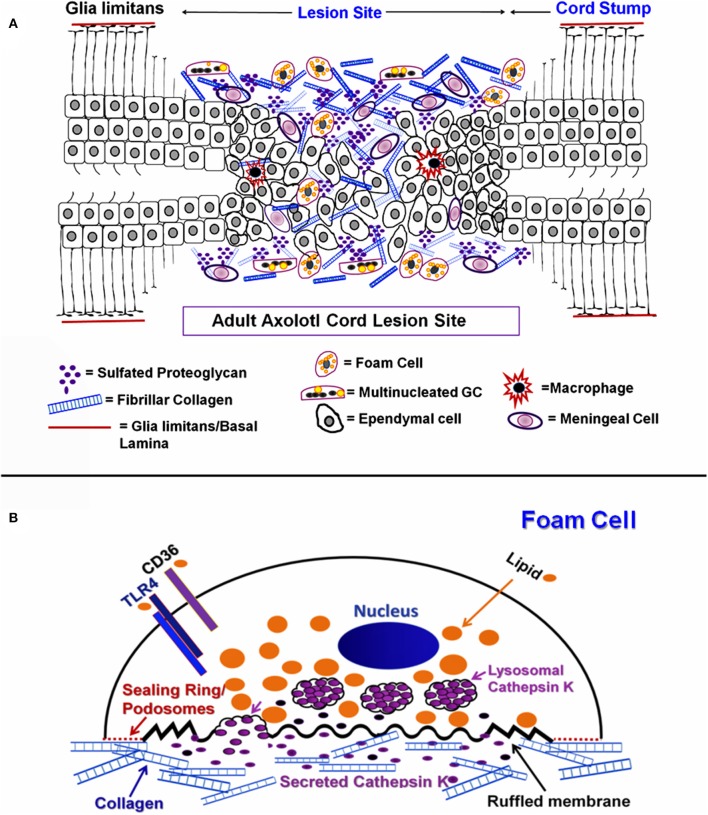
Diagram of ECM and cellular associations in regenerating cord. **(A)** Diagrammatic representation of mesenchymal ependymal cell outgrowth and meningeal invasion gap regeneration of the Axolotl cord lesion site. Ependymal cells, foam cells, MNGCs, macrophages and meningeal cells are shown within extracellular matrix. Neurons and oligodendrocytes are not represented. **(B)** Diagrammatic representation of a foamy macrophage producing cathepsin K.

The cell and ECM associations shown in Figures 1–3 are interpreted diagrammatically in Figure 15A. Fibrillar collagen from the meninges was found throughout the lesion site between the retracted spinal cord stumps after transection. Sulfated proteoglycan was found throughout the outgrowth, but concentrated closer to periphery. Meningeal cells invaded from the periphery while reactive ependymal cells withdrew their radial processes, became mesenchymal and migrated out into the lesion site (16). Macrophages and foamy macrophages were present.

Figure 15B shows a composite model of markers to be used throughout the experimental results, illustrating foam cell specializations that were characteristic to this cell type and are indicators of function within the Axolotl cord lesion site. On the unattached cell surface, is the lipid transporter CD36 which would mediate uptake of lipids, to be sequestered within the cytoplasm. TLR4/TLR6 acts in concert with CD36 in transport and are also known to be involved in fusion into MNGCs (23). The sealing ring structures and cathepsin K are show on and associated with the ECM-attached surface.

### Organization of the Lesion Site

There are species differences in urodele SCI responses, just as there are in rodents (10, 67). The ependymal outgrowth process during gap regeneration in newts is more in the form of an epithelioid bulb than mesenchymal outgrowth, but newt transection lesion sites share features with those seen here in the Axolotl (5, 12, 16, 68). In early stages of spinal cord regeneration in both *Notophthalmus viridescens* (Eastern Red-Spotted Newt) and *Ambystoma mexicanum* (Axolotl) a fibrillar collagen-rich ECM wraps the regenerating tissue (Zukor et al., Figure 7; our Figure 1E). In the Axolotl, fibrillar collagen-containing matrix encases and bridges the cut ends of the cord, and cells grow out into that material (Figures 1C,D). Zukor et al. (12) showed the accumulation of CSPG in the meninges after transection in the newt. The Axolotl ECM that supported foam cell, MNGC and ependymal cell invasion also contained sulfated proteoglycans (Figures 1E,F). In both the newt and Axolotl there is little sulfated proteoglycan present before injury (Figure 1B). Compared to mammalian SCI ECM, it still not entirely clear whether the interstitial matrix that forms with meningeal fibrosis in urodeles is intrinsically non-inhibitory in composition or organization, or whether regeneration proceeds because of successful removal of this material (69–71). Production of cathepsin K by the foamy macrophages, MMP and cathepsin production by ependymal cells, plus known MMP production by foamy macrophages in other tissues suggests that removal of the ECM material is likely a strong component of the process [Figure 11; (20, 72)].

Axolotl lesion site ECM appeared to have two zones: one closely wrapped around the regenerating cranial and caudal ends of the cord plus the material between the cut ends. Following fixation, they were separable, the wrapped material remained firmly attached to the regenerating cord (Figures 1C,E,F). Inflammatory response cells accumulate in this material in both the newt and Axolotl [(12); Figures 1–4; Supplemental Figure 2]. Though not explicitly identified, the newt TEM images shows lipid-laden cells among the immune response cells accumulated in the reactive meninges (12). In the Axolotl lesion site, the foamy macrophages were concentrated in and on the meninges of the regenerating cranial and caudal stumps (Figures 1, 2).

*In situ*, MNGCs are found in clusters on and in the fibrotic meninges (Figures 1G,H, 2D, 3A–C). It is not known if representative numbers of these cells are growing out of the lesion site explants *in vitro*, or whether they are so strongly attached to lesion site ECM that they are under-represented on the culture dish. Cluster of MNGCs are not seen in the culture dish outgrowth (Supplemental Figure 6). Further studies of axolotl meningeal MNGC formation and behavior are required.

### Identification of Ependymal Cells

In these culture conditions (+EGF, fibronectin coating), the cells that grow out of the lesion site explants are ependymal cells, foamy macrophages, MNGCs and, rarely, melanocytes [Figures 2E, 5–11, 13; Supplemental Figure 7; (6, 16, 56, 73)]. Other cord or meningeal cells fail to exit the explants. The Axolotl ependymal cells have been thoroughly characterized in prior marker studies. Intact Axolotl cord ependymal cells are cytokeratin and glial fibrillary acidic protein (GFAP)-positive and the cytokeratins and GFAP are lost during epithelial-mesenchymal transition (6, 16, 73). All of the injury-reactive Axolotl ependymal cells express the stem/progenitor cell marker mRNA-binding protein Musashi1 (16). A Musashi1 expression image is shown, again, at reviewer request along with GFAP intermediate filaments in the process of turnover. The appearance of the large ependymal nuclei is also quite distinctive (56). Examples of control culture Musashi1 localization and perinuclear GFAP *in vitro* are shown in Supplemental Figure 8.

### Identification of Axolotl Foamy Macrophages

The identification of the lipid-laden mononucleated cells in the Axolotl lesion site *in situ* and *in vitro* as foamy macrophages was based on systematic examination of markers used in mammalian foamy macrophages. The labeling of lipid droplets with Oil Red O was primarily perinuclear (Figure 5), as it was in mammalian foamy macrophages (29). The cells also took up DiI-Ox-LDL like mammalian foam cells [Figure 7; (28, 74)]. The lipid transporters localized in the Axolotl foamy macrophages were CD36 and TLR4, the primary transporter and co-transporter used by mammalian foamy macrophages [Figure 6; (75)]. An active role for CD36 in lipid transport was supported by the specific inhibition of Ox-LDL uptake by the modified fatty acid Sulfo-*N*-succinimidyl Oleate [Figure 8; (64)]. Axolotl spinal cord foamy macrophages took up myelin fragments *in vivo* and *in vitro*, like foam cells in diseases including multiple sclerosis, and within mammalian spinal cord after SCI [Figures 2C, 9, 10; (27, 34)]. Myelin uptake by macrophages in mammalian SCI and multiple sclerosis models is a phagocytic process, and membrane-bound myelin inclusions are seen in our foamy macrophages and smaller MNGCs by TEM [(41, 76); Figure 3C inset]. Axolotl foamy macrophages produced cysteine proteinase cathepsin K characteristic of mammalian foam cells, and cathepsin K was seen localized within sealing rings [Figure 11; (54)]. Finally, TEM examination shows ultrastructural features like a ruffled border and perinuclear intermediate filaments seen in mammalian foamy macrophages [Figures 3B,C; Supplemental Figure 2; (59)].

### Misidentification of Axolotl Foamy Macrophages

Macrophages have been described in injured salamander spinal cord, but not foamy macrophages (36). Results presented here (Figures 1B, 2C) showed that foamy macrophages were absent from distal stump and control Axolotl cord but accumulated in the fibrotic meninges within 1–1.5 mm on each side of the transection site. There is a history of noting, but not accurately identifying, these cells in the urodele regeneration literature. Our early studies show the presence of lipid-laden mono- and multinucleated cells in reactive axolotl cord ependymal cultures that were considered to be osteoclasts and their precursors that had migrated from neural arch following laminectomy performed to expose the cord for transection (56). Maier and Miller (77) noted the presence of what appear to be foamy macrophages among newt limb blastema cells in culture, but called them signet cells because they had eccentrically positioned nuclei. Washabaugh and Tsonis (78) identified these cells as signet cells following Maier and Miller [(78), Figure 1B] in regenerating newt limb blastema cultures, noting the presence of granules that appear to be lipid droplets. In a newt spinal cord regeneration study, Zukor et al. (12) showed macrophages and other white blood cells in and around the newt spinal cord lesion site by TEM, and some of these appear to have foamy cytoplasm [(12), Figures 7C,D]. The present studies are the first to show that these lipid-laden cells concentrate in and on the meninges at the lesion site in urodele spinal cord regeneration and to characterize them as foamy macrophages based on functional markers, lipid labeling and uptake studies.

### Possible Source of Foamy Macrophages

Two waves of macrophage recruitment have been described in mammalian SCI: first M1 (pro-inflammatory) macrophages of splenic origin, then M2 (anti-inflammatory) macrophages from either bone marrow or resident tissue immune cells (79). In a mouse spinal cord contusion system, M1 macrophages precursor cells were carried in the vasculature of the leptomeninges (mainly the arachnoid layer), which exit through the subarachnoid space to become M1 macrophages within the cord (80). Precursors to the M2 macrophages enter the brain ventricular-choroid plexus, travel through the cerebrospinal fluid, then invade the injured spinal cord and became M2 macrophages (80). It appears that conversion to mammalian cord foamy macrophages occurs within the spinal cord tissue (34, 35). Control Axolotl cord and stump tissue meninges have no foam cells (Figures 1B, 2C), but they are abundant in the lesion site. The source of these foamy macrophages and MNGCs is not yet known. Future studies will be required to determine whether the entire population is recruited from circulating monocytes and macrophages or if resident macrophages proliferate locally and invade the lesion site.

### Multinucleated Giant Cells

Activity differs between large and smaller MNGCs in our tissue culture system: smaller MNGCs and mononucleated foamy macrophages were more like each other than were large and small MNGCs. Cathepsin K activity, expression of the lipid transporter CD36 and co-transporter TLR4 were found in mononucleated foam cells and small MNGCs ( ≤ 6 nuclei, Figures 6E,G, 11E). TLR4 was absent from very large MNGCs (Figure 6G), consistent with the absence of lipid droplets. Very large MNGCs were generally lipid droplet free: one with 36 nuclei is shown in Figure 6G and Supplemental Figure 6D along with other non-lipid-containing MNGCs (Supplemental Figure 6), so their origin or metabolic state may be very different from the smaller lipid-laden MNGCs. Small MNGCs displayed cathepsin K activity (Figure 11E), but large MNGCs were not seen in any of the cathepsin K antibody labeled cultures, so the relationship of large numbers of nuclei and ECM proteolysis is not yet known in our system.

### Cytoskeleton Identity and Function

F-actin localization showed that the foamy macrophages (Figure 13D) and MNGCs (Figure 2D) attach to the reactive meninges with characteristic podosome-studded sealing rings. These integrin and actin-containing structures are present in other types of macrophages and osteoclasts, where they are associated with a polarized (substratum-attached vs. free surface) morphology needed for normal secretory and transport functions (24, 32).

TEM studies show Axolotl meningeal foamy macrophages and MNGCs with masses of perinuclear intermediate filaments (Figure 3C; Supplemental Figure 2B). Vimentin intermediate filament accumulation is characteristic of mononucleated osteoclast precursors, osteoclasts and osteoclast-like MNGCs in mammals and clustering of nuclei in MNGCs occurs within a netlike “nest” of vimentin intermediate filaments (59).

### Cathepsin K *in vivo* and *in vitro*

The cysteine protease cathepsin K is probably best known for its role in osteoclast pit production on bone (24, 32). Cathepsin K also performs a role in foamy macrophage and macrophage-derived MNGC ECM degradation (32, 52–55). Foamy macrophages in the Axolotl spinal cord lesion site showed cathepsin K localization (Figure 11A) and, *in vitro*, foamy macrophages and small MNGCs were all cathepsin K positive (Figures 11B,C,E). *In vitro*, cathepsin K was localized within the sealing ring between the cell and culture substrate and also within the cytoplasm of foamy macrophages (Figures 11B,C,E).

In the normal CNS, cathepsin K is expressed in mouse choroid plexus ependymal cells, but it is not a universal feature of intact ependymal cells throughout the mouse CNS (81). It is not known how widely distributed cathepsin K might be in the urodele CNS.

The activation of cathepsins is a complex process. Association of cathepsins with negatively charged glycosaminoglycan chains of proteoglycans allows autocatalytic activation (82). In regenerating urodele spinal cord there is abundant sulfated proteoglycan, including CSPG [Figure 1; (12)]. CSPG, specifically, has been shown to be involved in extracellular autoprocessing of pro-cathepsin K in osteoclasts (83), and is a good candidate for the same role in reactive Axolotl ependymal cells, foamy macrophages and MNGCs. This response to the fibrotic meningeal sulfated proteoglycan would mediate matrix degrading activity locally, contributing to prevention of permanent scar formation.

### Intracellular Distribution of Cathepsin K

Ependymal cell cathepsin K localization was markedly asymmetrical (Figures 11B,D,E) and could be related to leading edge/trailing edge polarity. In studies of tumor cells, related to understanding metastatic migration, attempts have been made to assign cathepsin localization to the leading edge or trailing edge of migrating cells. In stationary breast carcinoma cells, the localization of cathepsin B is perinuclear, but it is concentrated on one side of the nucleus in a moving cell (84). It is reported that this is the trailing edge of the cell (84). In a study of cathepsin H localization in a prostate cancer cell line, the cathepsin co-localizes with talin, a leading-edge protein associated with focal adhesions in cell migration (85). The cathepsin H was also abundant around the nuclei. The Jevnikar et al. (85) paper is the only one showing a leading edge or trailing edge marker (talin) co-localized with a cathepsin.

In our cultured foamy macrophages and MNGCs, the cathepsin K was localized either within the sealing ring (Figures 11B,C) or in a polarized fashion in elongated cells (Figures 11B,E). In primary T-lymphocytes and T-lymphocyte lines, cathepsin X is found strongly localized in the both the leading edge and in the uropod, the trailing, deadhesive structure of lymphocytes (86).

While associated with leading edge/trailing edge polarity morphologically, correlation of cathepsin localization with a mechanism of directional cell migration is not possible at this time in either the ependymal cells or foamy macrophages.

### Lipid in Ependymal Cells and Foamy Macrophages

TEM examination has shown that normal urodele ependymal cells contain some small lipid droplets. During regeneration, newt tail cord TEM examination shows the accumulation of lipid droplets in ependymal cells (2). The cytoplasm of intact mammalian ependymal cells label with DiI, but the magnification and resolution of the published images in that study do not permit identification of lipid droplets (87). In other mammalian studies, normal, young, non-pathological CNS ependymal cells do not contain stores of lipid. In the ventral portion of the brain, lateral ventricles of young mice stained with Oil Red O shows little lipid in the ependymal cells (88). There is more lipid in middle-aged mice and a high level of lipid in aged mouse ependymal cells (88). Similarly, there is a large increase of lipid in Alzheimer's Disease choroid plexus ependymal cells connected to expression of receptors for the transcytosis of LDL, lipoprotein receptor-related proteins-1 and 2 (megalin) (89, 90). So lipid accumulation in ependymal cells is associated with a disturbed or pathological state in ependymal cells.

The combined Oil Red O, DiI and DyRect labeling showed both neutral lipid and polar lipid content in both ependymal cells and foamy macrophages (Figure 5). The Axolotl ependymal cells and foamy macrophages also expressed the lipid scavenger receptor CD36 *in vivo* and *in vitro* indicating a common mechanism of lipid uptake (Figure 6). Therefore, both ependymal cells and foamy macrophages could be participating in removal of toxic lipids released or formed after neural injury. The source of neutral and polar lipids in both populations of cells could involve uptake of native and oxidized lipoproteins as well as myelin breakdown products from the Axolotl spinal cord lesion site.

### Autofluorescence

Autofluorescence in Axolotl foamy macrophages confounds the use of green fluorochromes, including the Dyrect neutral lipid fluorochrome (Supplemental Figure 4). The source is not necessarily unique, but foamy macrophages can take up lipofuscin (ceroids) in pathological conditions (Dvorak and Monahan-Early, 1992). Foam cells exposed to Ox-LDL accumulate lipofuscin, and lipofuscin autofluorescence has even been used as a marker for phagocytic CNS macrophages (74, 91). Lipofuscin can also be liberated by neuronal death. Autofluorescence of lipofuscin has an emission spectrum in the region of 430–490 nm, with a maximum in the yellow range (92). This would bleed through typical green fluorescence filters. The degree of autofluorescence of foamy macrophages and ependymal cells varies, suggesting differential uptake of autofluorescent compounds or differential exposure to them within the lesion site. Maier and Miller (77) noted the autofluorescence of what they termed “signet cells” from regenerating newt limb blastemas.

### Myelin Uptake

The myelin uptake behavior seen in the present study could be beneficial in regeneration. The axolotl form of the myelin-associated inhibitory molecules Nogo-A (axNogo), the Nogo receptor and myelin associated glycoprotein (MAG) are expressed in the Axolotl CNS, including during regeneration, suggesting that they are not inhibitory. Localization of axNogo and MAG in urodeles is primarily in gray matter neurons and ependymal cells, unlike the myelin/oligodendrocyte localization in mammals (93, 94). Despite these differences in the effects of myelin-associated molecules between mammals and urodeles, lesion site myelin was removed by ependymal cells and foamy macrophages during activity *in vivo* and *in vitro* (Figures 9, 10). One additional consideration regarding removal of myelin is the role this process plays in modulating M1 pro-inflammatory to M2 anti-infammatory phenotype of foamy macrophages in multiple sclerosis models (22, 27, 34, 95–97). Pro- or anti-inflammatory properties of Axolotl spinal cord foamy macrophages remain to be studied.

There were distinct differences in initial myelin content in ependymal cells placed in culture: some regions of explants outgrowth labeled heavily and some not at all. This could be dependent on their initial location within the lesion site. The foamy macrophages are concentrated around the regenerating ends of the cord, while the ependymal cells are growing out into this zone from the spinal cord stumps. In regions close to the transection site, the foamy macrophages may be the initial cell population to engulf myelin fragments, outcompeting the later-arriving ependymal cells. Deeper within the lesion site, where there are fewer foamy macrophages (Figures 2A,B), ependymal cells may be the primary cells to take up myelin.

### Are Some Cells “Full” of Ox-LDLIn Culture?

Lipid droplets are the most visible form of cellular lipid storage. A mature lipid droplet is described as a mass of neutral lipid within a single phospholipid leaflet membrane (98). Lipid droplets have a complex genesis and breakdown process within cells. Neutral lipids are generated enzymatically in the ER and can be trafficked to many parts of the cell including and the nucleus, lysosomes and vacuoles. Intracellular storage of lipids reflects a balance between uptake, consumption, interconversion of lipid forms and release (98, 99). Cellular stress and immune factors play significant roles in lipid balance (99).

The mechanisms underlying lipid balance in macrophages have been studied most extensively in non-neural diseases, such as atherosclerosis, non-alcoholic fatty liver disease (NAFLD) and pulmonary alveolar proteinosis (PAP), including the conversion of high levels of LDL to Ox-LDL related to foamy macrophage formation and function (29, 100). Ox-LDLs are not recognized by a macrophage's native LDL receptor, but they are recognized by the CD36 scavenger receptor (29, 75). Lipid uptake *via* scavenger receptors is not subject to feedback regulation and can lead to excessive accumulation of lipids in affected cells (75, 90, 101). Cholesterol esters are hydrolyzed enzymatically into free cholesterol, which can be utilized by cellular processes, exported from the cell, or converted back to cholesterol esters to prevent toxic effects from excess free cholesterol. Export of high-density lipoprotein is the primary means of lipid efflux from cells under normal conditions, though there is some passive free cholesterol efflux (29, 100).

Cholesterol ester hydrolysis may be actively inhibited by Ox-LDLs after prolonged exposure (102, 103). Exposure to Ox-LDLs can lead to lysosomal accumulation of lipids, rather than cytosolic accumulation of lipid droplets (102–104). However, there are species-specific differences in Ox-LDL response: free cholesterol and cholesterol esters were present in pigeon macrophage lysosomes after Ox-LDL exposure, while lipid accumulation was cytosolic in mouse macrophages (103). Lysosomal dysfunction caused by Ox-LDLs may not be easily reversed, which could explain why some of our foam cells don't take up much additional Ox-LDL *in vitro* (Figure 7). Other modified LDLs can be metabolized without adversely affecting essential lysosomal acidity and our foam cells are likely taking up many types of modified LDLs, but those with a high existing Ox-LDL burden would be inactive in the DiI-Ox-LDL uptake experiments (105, 106). Prolonged lysosomal dysfunction could be why our foam cells remain so full of lipid long after removal from the injury site. The absence of DiI-Ox-LDL uptake in ependymal cells *in vitro* (Figures 7A,B, 8A) could reflect natural uptake of less oxidized forms of LDL *in vivo*, or a greater tendency toward lysosomal dysfunction.

### ECM Degradation vs. Synthesis

#### ECM Degradation

Cells migrate into the Axolotl cord lesion site through a collagen- and proteoglycan-rich ECM (Figures 1D–F). The regenerating cord shows a massive increase in the amount of ECM investing the cord, compared with the normal cord meningeal ECM, with a disproportional increase in sulfated proteoglycan content (Figures 1B,E).

ECM is known to be phagocytosed and degraded intracellularly by a variety of amphibian cell types, including anuran (frog and toad) macrophages during the ECM turnover associated with metamorphosis (107). TEM studies, here, show foam cells are attached to, and embedded in, fibrillar collagen (Figures 3B,D). TEM examination shows phagocytosed fibrillar collagen in a foamy MNGC (Figure 3D; Supplemental Figure 2A). In an enlargement of Figure 3B (Supplemental Figure 2A) interstitial fibrillar collagen can be seen among a group of ependymal cells, but none of them showed phagocytosed collagen. Either phagocytosis of the fibrillar collagen is accomplished only by the foamy macrophages, or reactive ependymal cell collagen uptake is in the form of smaller peptides resulting from extracellular degradation. In Figure 3F an image from a site in a zone farther from the transection site where the interstitial collagen has already been removed: no intracellular collagen was seen.

The ependymal cathepsin K production described here, and ependymal MMP production characterized previously in Axolotl cord regeneration, show that ependymal cells can participate actively in ECM degradation mediated by secreted proteases [Figure 11; (20)]. Foamy macrophages are known to produce MMP9 and cathepsin K in diseases including atherosclerosis, and MMP9 in multiple sclerosis (53, 108, 109).

Axolotl foamy macrophages and smaller MNGCs participate in ECM removal by secretion of cathepsin K as well as phagocytosis (Figures 3D, 11A–C,E; Supplemental Figure 2A). MNGCs are seen in or on the reactive meninges (Figures 1G,H, 2D, 3A,B) and are known to participate in ECM degradation in other system including MNGC tumors (32, 52). In our primary tissue culture system, smaller MNGC actively produce cathepsin K, while all of the foam cells do so (Figure 11). A full understanding of the ECM proteolytic repertoire of cord lesion site MNGCs will require much larger numbers in future studies involving stimulated fusion *in vitro*.

#### ECM Synthesis

The participation of ependymal cell endfeet in reforming the *glia limitans* has long suggested that they are producing ECM in later stages of the regeneration process (2). The expectation regarding ECM production by meningeal foam cells is not at all clear. In a few experimental disease-related models, a fibrotic role has been indicated for macrophages. This includes exposure of human macrophages to native LDL which stimulates proteoglycan secretion, a phenomenon with implications for vascular wall trapping of apolipoprotein B-containing lipoproteins in atherosclerotic plaques (110). This phenomenon may be related to the production of a proteoglycan form of macrophage colony stimulating factor in atherosclerotic plaque macrophages (111). In mice with surgical sponge-induced granulomas, and in atherosclerotic plaques, a variety of genes associated with fibrosis are expressed in transcriptome analysis: several collagen peptides and the small proteoglycans decorin and biglycan (112). This expression is higher in foamy macrophages vs. non-foamy macrophages (112).

The *in vitro* ^3^H-glucosamine uptake results presented here suggest that ependymal cells are producing glycosaminoglycans and proteoglycans during the ependymal outgrowth process, depositing new ECM even as they are engaging in lesion site matrix turnover (Figures 14A,C). The foamy macrophages, however, show no evidence of ECM production from the ^3^H-glucosamine uptake studies (Figures 14B,C).

### Foamy Macrophage/Ependymal Interactions: Beneficial to Regeneration or Not?

In mammalian SCI, macrophages support scar formation and inhibit axonal regrowth (55, 113, 114). Macrophages are required in Axolotl limb and heart regeneration, so there is evidence that innate immune system cells can have a positive role in urodele regeneration (115, 116). In mouse heart, this positive role for macrophages does not extend beyond the neonatal stage (117).

Less is known about macrophages and macrophage/target cell interactions in urodele spinal cord regeneration. A study using lectin probes shows macrophages within the injured Axolotl spinal cord (36). In the newt studies by Zukor et al. (12), TEM examination shows macrophages in contact with neurons in an early stage of axonal regrowth called “wisping,” as well as being present in the reactive meninges (12). In the Zukor et al., Figure 7D, a lipid-laden macrophage that could be a foam cells is shown in contact with an axon, and foam cell-like white blood cells are shown in the reactive meninges (12), Figure 7C. Though not identified as such, foamy macrophages may be present in the regenerating newt cord.

The present study examined the interaction between foamy macrophages and ependymal cells (Figures 13, 14). Mononucleated foamy macrophages were abundant in Axolotl cord lesion site tissue *in situ* and *in vitro* (Figures 2B,E, 6C, 13C). Statistical analysis showed a highly significant association of the presence of foamy macrophages with an increased degree of dispersal of ependymal cells *in vitro* (Figures 2E, 5A,C, 13, 14). It is not known whether the interaction involves cell-cell contact or secreted factors.

The co-migration of foamy macrophages and ependymal cells was strong during the period of adult Axolotl cord gap regeneration corresponding to mesenchymal ependymal outgrowth (2 weeks, Figures 13A–D), while at 4 weeks of regeneration, when the ependymal cells from cranial and caudal stumps have reconnected, the foam cells were no longer present (Figure 13E). It is not yet known whether the foamy macrophages and MNGCs are excluded to the periphery with the meninges, undergo cell death or both.

The position of these cells on and within the reactive meninges investing the regenerating stumps suggests a model in which the foam cells and smaller MNGCs act on the invasive meninges from the “outside-in,” while the ependymal cells act from within the cord into the lesion site to remove fibrotic meningeal ECM [Figures 1E–H, 2A,B, 15A; (16, 20)]. Because they are attached to and within the invasive meninges, the foamy macrophages are concentrated in the lesion site distal to the zone of ependymal outgrowth. The ependymal cells grow out to meet and mix with the foamy macrophages.

Foamy macrophages can release growth factors, such as FGF2 and TGFβ, that are bound by ECM (118). Decellularized human meninges retains FGF2 and VEGF, suggesting that the release of ECM-bound growth factors by the foamy macrophages proteases could be of importance in the Axolotl cord lesion site (119). Urodele ependymal cells respond to TGFβ and FGF2 (6, 120).

The relationship of foamy macrophages to ependymal outgrowth, and their known activity, places the macrophages in a position to open the way for ependymal cells through ECM *in vitro* during ependymal outgrowth. In combination with the ECM degrading and synthetic capacity of the reactive ependymal cells, this juxtaposition of the foamy macrophages and ependymal cells could help maintain directional outgrowth across the lesion site.

## Data Availability Statement

The datasets generated for this study are available on request to the corresponding author.

## Ethics Statement

The animal study was reviewed and approved by IUPUI School of Science Institutional Animal Care and Use Committee (SARC).

## Author Contributions

NE, HT, HS, ME, TB-A, and EC contributed the conception and design of the study. SS developed the wholemount staining procedures and their application to this study. TB-A performed the statistical analysis. ME and DS performed the TEM analysis and interpretation. DS planned and performed the examination of the time course of meningeal regeneration. DS-B provided the immunological interpretation essential for experimental design. MK developed and applied the wholemount actin organization studies. TB-A, EC, and DS-B developed the diagrammatic interpretation of the lesion site, foamy macrophages, and MNGCs. EC, TB-A, and NE wrote, and EC and TB-A coordinated the revisions to the manuscript. HT and HS initiated this project as students in the laboratory of EC.

### Conflict of Interest

The authors declare that the research was conducted in the absence of any commercial or financial relationships that could be construed as a potential conflict of interest.
